# Loss of KDM6A confers drug resistance in acute myeloid leukemia

**DOI:** 10.1038/s41375-019-0497-6

**Published:** 2019-06-14

**Authors:** Sophie M. Stief, Anna-Li Hanneforth, Sabrina Weser, Raphael Mattes, Michela Carlet, Wen-Hsin Liu, Michael D. Bartoschek, Helena Domínguez Moreno, Matthias Oettle, Julia Kempf, Binje Vick, Bianka Ksienzyk, Belay Tizazu, Maja Rothenberg-Thurley, Hilmar Quentmeier, Wolfgang Hiddemann, Sebastian Vosberg, Philipp A. Greif, Klaus H. Metzeler, Gunnar Schotta, Sebastian Bultmann, Irmela Jeremias, Heinrich Leonhardt, Karsten Spiekermann

**Affiliations:** 1 Department of Medicine III, University Hospital, LMU Munich, Munich, Germany; 2 German Cancer Consortium (DKTK), Heidelberg, Germany; 3 German Cancer Research Centre (DKFZ), Heidelberg, Germany; 4 Department of Apoptosis in Hematopoietic Stem Cells (AHS), Helmholtz Zentrum München, Munich, Germany; 5 Department of Biology II and Center for Integrated Protein Science Munich (CIPSM), Human Biology and BioImaging, LMU Munich, Planegg-Martinsried, Germany; 6 Biomedical Center and Center for Integrated Protein Science Munich, LMU Munich, Martinsried, Germany; 7 Department of Human and Animal Cell Lines, Leibniz-Institute DSMZ-German Collection of Microorganisms and Cell Cultures, Braunschweig, Germany; 8 Department of Pediatrics, Dr. von Hauner Children’s Hospital, LMU Munich, Munich, Germany

**Keywords:** Acute myeloid leukaemia, Cancer therapeutic resistance

## Abstract

Acute myeloid leukemia (AML) is an aggressive hematologic neoplasm resulting from the malignant transformation of myeloid progenitors. Despite intensive chemotherapy leading to initial treatment responses, relapse caused by intrinsic or acquired drug resistance represents a major challenge. Here, we report that histone 3 lysine 27 demethylase *KDM6A* (*UTX*) is targeted by inactivating mutations and mutation-independent regulation in relapsed AML. Analyses of matched diagnosis and relapse specimens from individuals with *KDM6A* mutations showed an outgrowth of the *KDM6A* mutated tumor population at relapse. KDM6A expression is heterogeneously regulated and relapse-specific loss of KDM6A was observed in 45.7% of CN-AML patients. *KDM6A*-null myeloid leukemia cells were more resistant to treatment with the chemotherapeutic agents cytarabine (AraC) and daunorubicin. Inducible re-expression of KDM6A in *KDM6A*-null cell lines suppressed proliferation and sensitized cells again to AraC treatment. RNA expression analysis and functional studies revealed that resistance to AraC was conferred by downregulation of the nucleoside membrane transporter *ENT1* (*SLC29A1*) by reduced H3K27 acetylation at the ENT1 locus. Our results show that loss of KDM6A provides cells with a selective advantage during chemotherapy, which ultimately leads to the observed outgrowth of clones with *KDM6A* mutations or reduced KDM6A expression at relapse.

## Introduction

Acute myeloid leukemia (AML) is characterized by expansion of abnormal myeloid precursor cells in the bone marrow and blood. If not treated, AML can progress quickly and become fatal within a few months. Standard strategies for initial therapy, which have not changed substantially during the last 30 years [1], include cytarabine (AraC) in combination with anthracyclines like daunorubicin (DNR). Although the majority of AML patients achieve complete remission following induction chemotherapy, the disease reoccurs in 60–65% of younger adult patients (≤60 years) within 3 years after diagnosis [2]. Relapse represents the major cause for treatment failure and drug resistance is likely to play a role in its development. Recently, we have analyzed paired diagnosis and relapse samples of 50 cytogenetically normal (CN) AML patients and found *KDM6A* as a novel relapse-associated gene in AML [3]. KDM6A (or UTX) is a JmjC domain containing histone H3 lysine 27 (H3K27)-specific demethylase [4, 5] and belongs to the KDM6 family that include *KDM6B* and *UTY* [4, 6]. KDM6A can facilitate gene activation through the catalytic JmjC domain and is also a component of the COMPASS-like complex, which is important for chromatin enhancer activation [7–11]. *KDM6A* is frequently targeted by somatic loss-of-function mutations in cancer [12–15] including leukemia [16–18]. Dependent on the cancer type, KDM6A appears to possess distinct tumor-suppressive functions. In T-cell acute lymphoblastic leukemia (T-ALL), *KDM6A* mutations are located almost exclusively in the JmjC domain [16, 17] and inactivation of the single *KDM6A* copy in males is sufficient to contribute to T-ALL pathogenesis [17]. In contrast, hematopoietic-specific loss of *Kdm6a* induces leukemogenesis through demethylase-independent alterations in H3K27 acetylation, H3K4 monomethylation and chromatin accessibility [19].

Using diagnosis and relapse samples from AML patients, patient-derived xenografts (PDX), and leukemia cell lines, we investigated the status of KDM6A during disease progression and the impact of KDM6A loss on chemotherapy resistance. We found three AML patients with enrichment of *KDM6A* loss-of-function mutations at relapse and relapse-specific loss of KDM6A mRNA and protein expression in 45.7% of CN-AML patients and 44.4% of AML patients, respectively. Reduction or loss of KDM6A expression in myeloid cell lines leads to increased resistance towards AraC and DNR treatment. Whereas re-expression of KDM6A in *KDM6A*-null cell lines sensitizes cells to AraC treatment. AraC resistance is achieved by reduction of the drug influx transporter ENT1. Taken together, our findings highlight KDM6A as a novel mediator of drug resistance in AML.

## Materials and methods

### Cell culture

Cell lines (Supplementary Table 1) were obtained from DSMZ (Braunschweig, Germany) and cultured according to the supplier’s recommendation. PDX AML samples were recovered from mice and cultured as previously described [20]. Authentication of cell lines was performed using short tandem repeat typing. Testing for Mycoplasma contamination was done using the MycoAlert Mycoplasma detection kit (Lonza).

### Patients

Our analysis was based on samples from AML patients from the AMLCG-99 trial (NCT00266136), AMLCG-2008 trial (NCT01382147), and the Department of Medicine III, University Hospital, LMU. Informed consent for scientific use of sample material was received from all study participants in accordance with the Declaration of Helsinki.

### Proliferation assay

Cells were treated with cytarabine (Selleck Chemicals, Houston, TX, USA), DNR (in-house), 6-thioguanine (Sigma Aldrich), or NBMPR (Sigma Aldrich). After 72–96 h, viable cells were counted on Vi-Cell Cell Viability Analyzer (Beckman Coulter, Krefeld, Germany). For long-term proliferation, cells were treated three times (d0, d4, d8) and viable cells were counted every second day. Unpaired, two-tailed Student’s *t*-test and calculation of IC_50_ values were performed using GraphPad Prism version 6.07 (GraphPad Software, La Jolla, CA, USA). PiggyBac KDM6A cells were cultured +/− doxycycline (0.5 μg/mL) for 48 h followed by treatment with AraC +/− doxycycline for 72 h. For longer proliferation, cells were cultured +/− doxycycline (0.5 μg/mL) for 8 days. Every 2 days, cells were counted and cultured in fresh medium +/− doxycycline.

Additional methods are provided in supplementary methods.

## Results

### Gain of *KDM6A* mutations at relapse

**Fig. 1:**
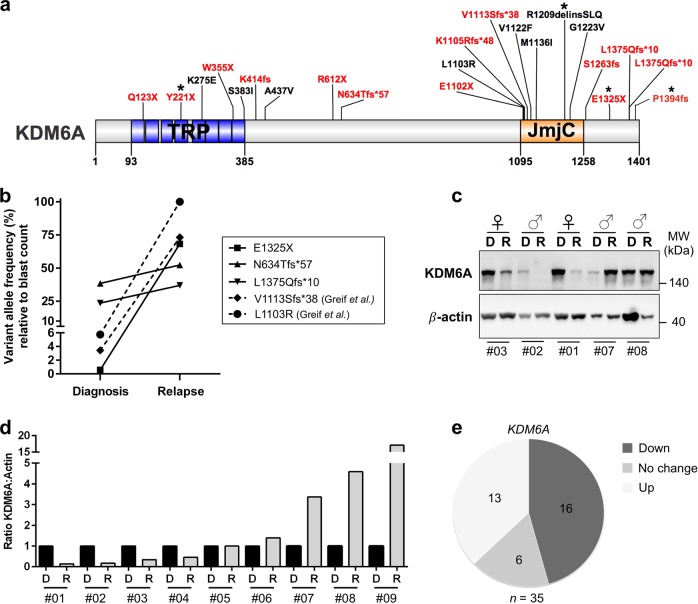
Gain of recurrent *KDM6A* mutations at relapse and change in KDM6A RNA and protein expression at relapse. **a** Schematic overview of KDM6A protein structure (NP_066963.2) and mutations (red = truncating; black = missense) identified at diagnosis in 20 AML patients, illustrated using IBS software [40]. Location of mutations is displayed and amino-acid positions are indicated below the graph. Asterisk (*) signifies two patients harboring two mutations each. Presented *KDM6A* mutations are from AMLCG-99 trial (NCT00266136), AMLCG-2008 trial (NCT01382147), a CN-AML diagnosis-relapse cohort [3] and this work. TRP tetratricopeptide repeat, JmjC Jumonji C. **b** Comparison of variant allele frequency (VAF) between diagnosis and relapse in 5 AML patients with *KDM6A* mutations. Due to variations in blast count, VAF was calculated relative to the respective blast count. Raw data for mutation L1130R and V1113Sfs*38 originate from our previous study [3]. **c**, Immunoblotting for KDM6A expression in five AML patients at diagnosis (D) and relapse (R). Their respective gender is shown on top and the UPN is displayed below. MW, molecular weight; β-actin, loading control. **d** Comparison of KDM6A protein expression in nine AML patients without *KDM6A* mutations at diagnosis and relapse. The ratio of KDM6A to β-actin expression is displayed. Respective values at relapse were normalized to the corresponding diagnosis sample. **e** Pie chart illustrating the regulation of *KDM6A* mRNA expression in 35 CN-AML patients. The three groups, *KDM6A*-up, *KDM6A*-down and *KDM6A*-no change were defined as a change in expression between diagnosis and relapse of above or below 20%, respectively

Despite their initial response to chemotherapy, the majority of AML patients will develop chemotherapy resistance and relapse. Acquired *KDM6A* mutations were reported at relapse [3] pointing towards a novel mechanism of resistance in AML. To get insight into the biological relevance of *KDM6A* mutations, we first analyzed their locations in 20 AML patients at diagnosis. Patients with *KDM6A* mutations were from the AMLCG-99 trial (*n* = 6), AMLCG-2008 trial (*n* = 9/664), a diagnosis-relapse cohort (*n* = 2/50) [3] and this work (*n* = 3). We found a broad location pattern with the majority of mutations either located at or within the proximity of the tetratricopeptide repeat (TRP) or the JmjC domain (Fig. 1a). 65% of patients harbor either frameshift insertions/deletions or nonsense mutations suggesting a loss-of-function phenotype. In the majority of patients (12/20), the mutation occurred only in a subpopulation of AML cells, with a variant allele frequency (VAF) below 15% (Supplementary Fig. 1a). In addition to two previously described CN-AML patients [3], we identified three patients with recurrent *KDM6A* mutations using matched diagnosis and relapse samples, which were available for 3/18 patients (Fig. 1b; Supplementary Fig. 1b–d). In all patients we observed an increase in VAF of *KDM6A* mutations at relapse (Fig. 1b). The mutant clone E1325X showed the most striking increase at relapse (68.2% VAF), as it was barely detectable at diagnosis (0.58% VAF). Transplantation of relapsed tumor cells from this patient into immunodeficient mice (PDX model [20]) resulted in stable regeneration of *KDM6A* E1325X mutant clone (PDX AML-393; Supplementary Fig. 1b), which was verified by Sanger sequencing (Supplementary Fig. 1e). A second *KDM6A* mutation, P1394fs, was present in the same diagnosed patient with a 12.8-fold greater VAF (8.1%) than E1325X, but was lost at relapse (Supplementary Fig. 1b).

Next, we compared KDM6A expression in matched diagnosis and relapse samples of 9 AML patients (listed in Supplementary Table 2) with no detectable *KDM6A* mutation (Fig. 1c, d; Supplementary Fig. 1f). A strong decrease in KDM6A protein expression at relapse was observed in four patients whereas three patients showed increased expression at relapse. Additional analysis of *KDM6A* mRNA regulation in 35 CN-AML patients revealed a downregulation of *KDM6A* in 45.7% of patients (*n* = 16/35) and an upregulation in 37.1% of patients (*n* = 13/35; Fig. 1e and Supplementary Fig. 2). Interestingly, DNA methylation levels of KDM6A varied between AML patients at diagnosis and AML patients with high DNA methylation levels of KDM6A (top 25%) showed significantly shorter overall survival in a publicly available dataset [21] (Supplementary Fig. 3).

**Fig. 2:**
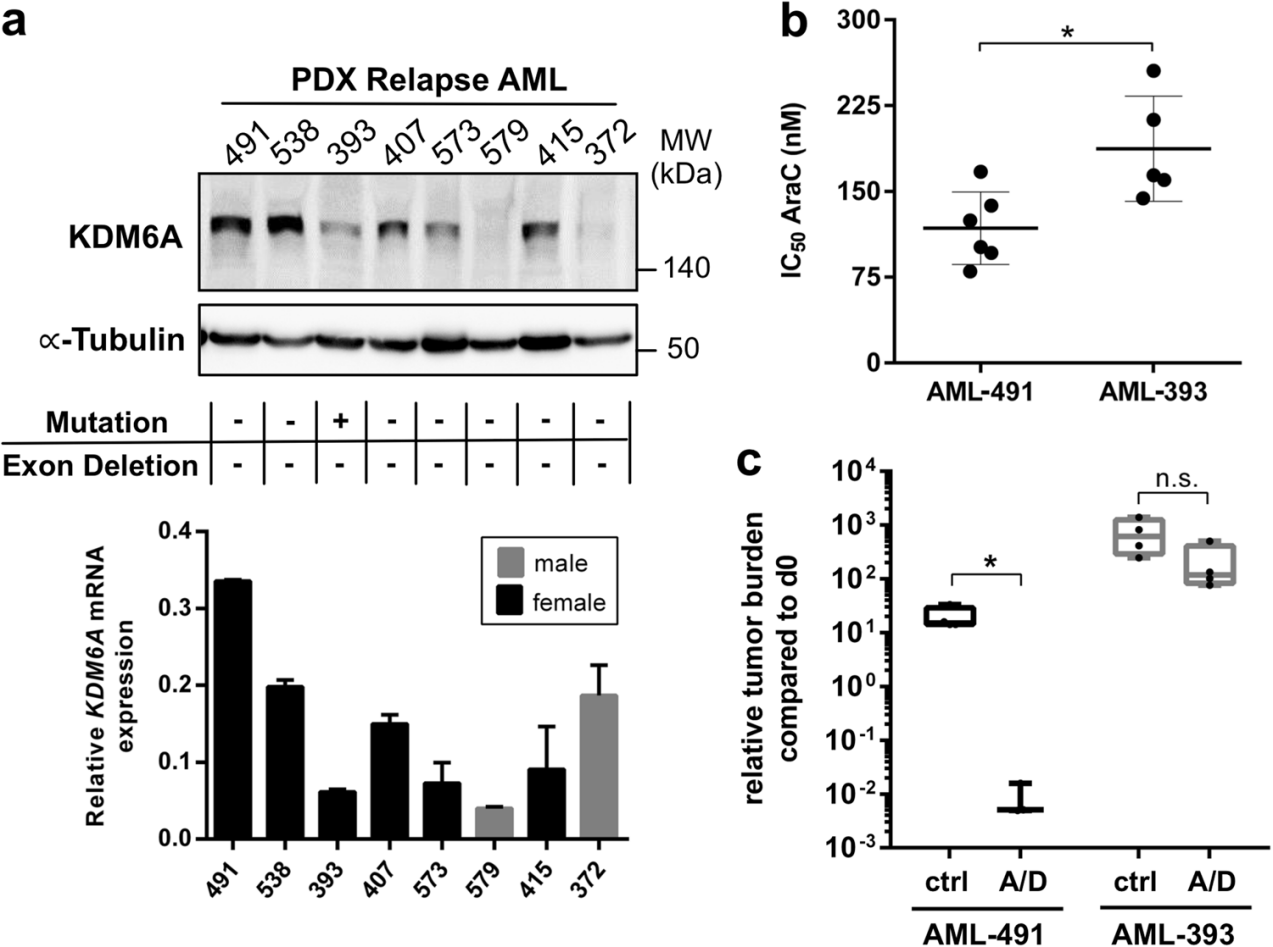
Heterogeneous KDM6A expression in relapsed PDX AML cells and decreased drug sensitivity in *KDM6A* mutant PDX AML-393 cells. **a** KDM6A mRNA and protein expression was analyzed in eight PDX samples from relapsed AML patients. For Western Blot, α-Tubulin was used as a loading control. *KDM6A* mRNA expression is shown relative to *GAPDH* expression. Male samples are highlighted as gray bar plots. The occurrence of *KDM6A* mutations or exon deletions is indicated with the “+” symbol. **b** Comparison of AraC IC_50_ values between *KDM6A* WT PDX AML-491 and *KDM6A* mutant PDX AML-393 in vitro. Mean ± s.d. are given for at least five independent experiments performed in duplicates. Unpaired, two-tailed Student’s *t*-test; *P = 0.016. **c** Comparison of tumor load reduction under in vivo chemotherapy in AML-491 and AML-393 bearing animals. Tumor burden was quantified by bioluminescence before (d0) and after (d28) two cycles of treatment with AraC (days 1–4, 14–17) and DNX (days 1, 4, 14, and 17) (A/D) or control treated animals (ctrl). Relative tumor burden at day 28 compared to d0 was calculated. Unpaired, two-tailed Student’s *t*-test; *P = 0.0157; n.s., not significant

To further investigate the relevance of KDM6A at relapse, we analyzed KDM6A mRNA and protein expression in 8 PDX AML samples, established from primary patients′ cells at relapse. 50% of PDX AML samples showed low or undetectable protein expression, which, except for AML-372, correlated with mRNA expression (Fig. 2a). Mutational analysis by MLPA and targeted sequencing revealed only the above described *KDM6A* mutation (E1325X) in PDX AML-393 (Supplementary Fig. 1b). No additional *KDM6A* exon deletion mutations were detected (Supplementary Fig. 4). mRNA expression of the histone demethylase *KDM6B* and the histone methyltransferase *EZH2* were slightly increased in AML-579 cells, whereas AML-538 showed low *KDM6B* and AML-491 low *EZH2* mRNA expression (Supplementary Fig. 5a, b). Analysis of the mRNA expression of *UTY* in PDX AML samples showed normal *UTY* levels (Supplementary Fig. 6d). Since we were unable to detect a low molecular weight band corresponding to the premature stop mutation E1325X (estimated protein weight: 145 kDa) in the female PDX AML-393 cells, we treated these cells in vitro with the proteasomal inhibitor MG132. No increase in overall KDM6A expression, but also no appearance of an additional band corresponding to E1325X was observed (Supplementary Fig. 5c), which might point towards a nonsense-mediated mRNA decay. When we overexpressed E1325X, L1103R, and V1113Sfs*38 *KDM6A* mutants in HEK293T cells, proteasomal inhibition resulted in a significantly elevated expression of these mutants whereas wildtype (WT) and the demethylase-dead mutant H1146A did not change (Supplementary Fig. 5d, e). These results suggest that reduction of KDM6A expression might be either facilitated by nonsense-mediated mRNA decay or by proteasomal degradation.

Next, we asked whether drug resistance might have resulted in the outgrowth of the *KDM6A* mutated population at relapse. Since AraC was a component of the induction therapy, we investigated whether *KDM6A* mutant cells are less sensitive to AraC treatment. Therefore, we used PDX AML models of the same adverse ELN classification [20] with (i) *KDM6A* WT (AML-491) and (ii) *KDM6A* mutant (AML-393) and compared the half-inhibitory concentration (IC_50_) of AraC. In vitro treatment for 72 h revealed a 0.63-fold decreased sensitivity towards AraC treatment for *KDM6A* mutant AML-393 compared to *KDM6A* WT AML-491 cells (187.3 nM vs. 117.9 nM; Fig. 2b). We observed the same effect in vivo after treating mice bearing AML-491 or AML-393 with two cycles of AraC and DaunoXome (DNX; liposomal DNR; Fig. 2c). Treatment dramatically decreased the tumor burden in *KDM6A* WT AML-491 bearing mice compared to control (*P* = 0.0157; Fig. 2c), whereas only a modest drop in tumor burden was observed in treated AML-393 bearing mice. Overall, these results indicate that a reduced KDM6A expression is associated with a decreased AraC sensitivity.

### Decreased AraC sensitivity in *KDM6A* mutant cells

**Fig. 3:**
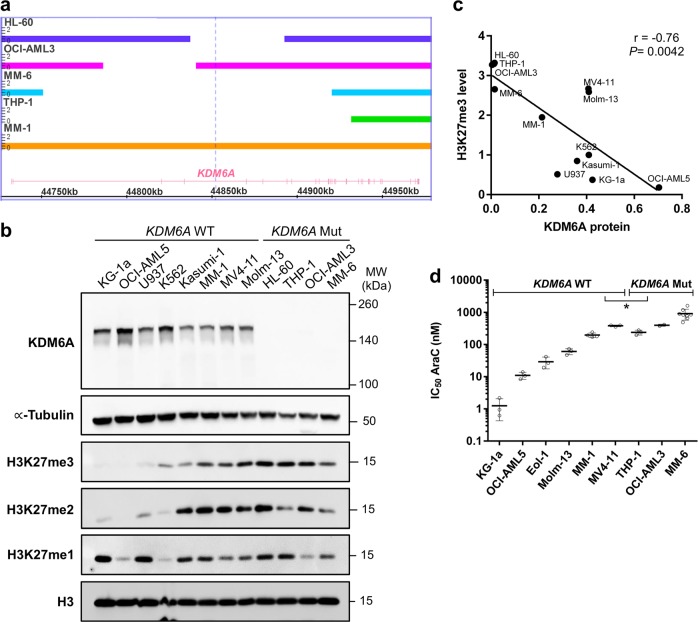
AML cell lines with KDM6A loss show high H3K27 tri-methylation and increased AraC resistance. **a** Identification of *KDM6A* aberrations in AML cell lines HL60, OCI-AML3, MM-6, and THP-1. MM-1 serves as a WT control. Bar thickness ranging from 0 to 2 indicates the copy number (CN) status. Haploidy (CN = 1) in cell lines from male patients is due to the X-chromosomal localization of *KDM6A*. HL-60, the only cell line derived from a woman has lost one of its X chromosomes. **b** Western blot for KDM6A expression and global H3K27 mono-, di- and tri-methylation levels in *KDM6A* WT and mutant human leukemia cell lines. α-Tubulin and total H3 were used as loading controls. Blots are representative of three independent experiments. MW, molecular weight. **c** Negative correlation between KDM6A protein expression and global H3K27me3 level in *KDM6A* WT and mutant human leukemia cell lines (Pearson’s correlation; *r* = −0.76, *P = 0.0042). Mean values of three independent analyzes are shown. **d** Comparison of AraC IC_50_ values between *KDM6A* WT and mutant male AML cell lines. To determine their respective IC_50_ values, AML cell lines were treated with increasing concentrations of AraC for 72 h. Mean of IC_50_ values from at least three independent experiments ± standard deviation (s.d.) are shown. Unpaired, two-tailed Student’s *t*-test; *P = 0.044

*KDM6A* exon deletion mutations have been observed in AML cell lines, MONO-MAC-6 (MM-6) and THP-1 [12]. To identify the frequency of *KDM6A* deletions in leukemia, we performed MLPA analysis for the *KDM6A* gene in 40 myeloid leukemia cell lines (Supplementary Table 1). We identified two additional AML cell lines, OCI-AML3 and HL-60, with in-frame deletions in exon 3–4 and 5–6, respectively (Supplementary Fig. 4). These deletions were confirmed by independent CytoScan HD Array hybridization analysis (Fig. 3a). Both in-frame deletions lead to a truncated protein of approximately 147 kDa (WT: 154 kDa). Although low to intermediate mRNA expression was detectable in the mutant cell lines (Supplementary Fig. 6a), KDM6A protein expression was completely absent (Fig. 3b). *KDM6A* mutant cells showed increased H3K27 tri-methylation, whereas H3K27 di- and mono-methylation levels were similar between WT and mutant cells (Fig. 3b). Global H3K27me3 was inversely correlated with KDM6A levels (r = −0.76; *P* = 0.0042; Fig. 3c) indicating a KDM6A dependent epigenetically altered phenotype. Additionally, we analyzed expression of *KDM6B* and *EZH2* via qPCR as these genes might compensate for KDM6A loss. For both genes, mRNA expression was similar between mutant and WT cells (Supplementary Fig. 6b, c). Analysis of *UTY* mRNA expression showed loss of *UTY* mRNA expression in two *KDM6A* mutant and three *KDM6A* WT cell lines (Supplementary Fig. 6d).

Next, we investigated whether KDM6A loss leads to increased AraC resistance. *KDM6A* mutant cells showed a trend towards higher AraC IC_50_ values compared to WT (Supplementary Fig. 7a). To eliminate gender-specific effects of *KDM6A* WT cells (higher expression in females as *KDM6A* escapes X inactivation [3, 17]), we compared the IC_50_ values of male AML cell lines. Male *KDM6A* mutant AML cell lines had significantly increased IC_50_ values compared to WT cells (*P* = 0.0441; Fig. 3d). We demonstrated previously [3] that MM-1 cells (*KDM6A* WT) are more sensitive to AraC treatment than the *KDM6A* mutant sister cells MM-6. To investigate if MM-6 also has a competitive growth advantage compared to MM-1 under AraC therapy, we performed a competitive assay mixing MM-1 with MM-6 cells in a 9:1 ratio. Native conditions as well as treatment with AraC significantly increased the number of MM-6 cells to 32.3% (native conditions) or 52.6% (270 nM AraC) after 9 days (Supplementary Fig. 7b). Treatment of MM-1 cells with AraC, DNR or 6-TG for 72 h, applying concentrations in the range of their respective IC_50_ values, induced an upregulation of KDM6A protein expression (Supplementary Fig. 8).

### Knockdown of *KDM6A* confers decreased AraC and DNR sensitivity in K562 cells

**Fig. 4:**
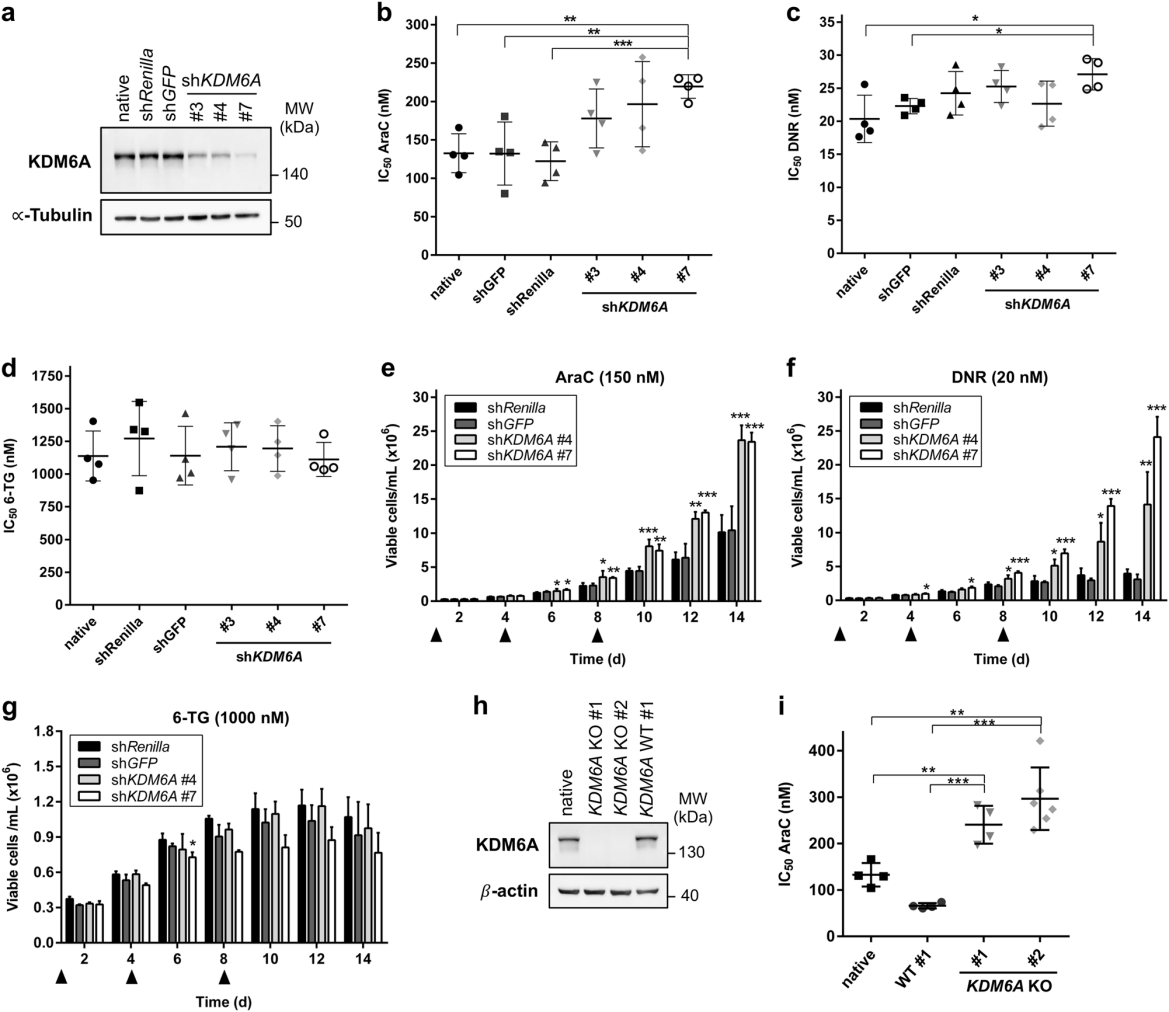
Reduction or loss of KDM6A expression confers decreased AraC and DNR sensitivity in K562 cells. **a** Immunoblot showing knockdown (KD) of KDM6A expression in K562 cells lentiviral transduced with three different shRNAs against *KDM6A*. sh*Renilla* and sh*GFP* serve as controls. Blot is representative of three independent experiments. MW molecular weight, α-Tubulin loading control. **b**–**d** Comparison of IC_50_ values for AraC (**b**), DNR (**c**), and 6-TG (**d**) between control and *KDM6A* KD in K562 cells. Cells were treated for 72 h with the respective drug. Mean of IC_50_ values from four independent experiments ± s.d. are shown. Unpaired, two-tailed Student’s *t*-test; *P < 0.05; **P < 0.01; ***P < 0.001. **e**–**g** Long-term proliferation assay measuring the amount of viable K562 cells, sh*Control* and sh*KDM6A*, every 2 days for 14 days in total. Cells were treated with 150 nM AraC (**e**), 20 nM DNR (**f**), or 1000 nM 6-TG (**g**) on Day 0, 4, and 8 as indicated with the triangle. Mean ± s.d. are given for three independent experiments. Unpaired, two-tailed Student’s *t*-test; *P < 0.05; **P < 0.01; ***P < 0.001. **h**, Immunoblot showing loss of KDM6A protein in *KDM6A* knockout (KO) K562 cells. Results of one representative experiment are shown (*n* = 3 experiments). MW molecular weight, β-actin loading control. **i** Increase in AraC IC_50_ values in both *KDM6A* KO K562 clones compared to WT #1 clone or native cells. Mean of IC_50_ values ± s.d. from at least four independent experiments are shown. Unpaired, two-tailed Student’s *t*-test; **P < 0.01; ***P < 0.001

Although AraC was part of the induction regimen in the five investigated patients with *KDM6A* mutations, the composition of induction therapy varied between patients and in certain cases also included the drugs DNR and/or 6-thioguanine (6-TG). To investigate if reduced expression of KDM6A leads to increased resistance towards multiple drugs, we performed lentiviral shRNA-mediated knockdown (KD) of *KDM6A* in the myeloid cell line K562. Of several tested shRNAs, sh*KDM6A* #3, #4, and #7 decreased KDM6A expression by 70% (#3, #4) or 90% (#7; Fig. 4a). Next, *KDM6A* KD or control cells were treated for 72 h with AraC, DNR, or 6-TG. *KDM6A* KD cells displayed decreased sensitivity towards AraC treatment (Fig. 4b) applying doses within the range of reported AraC plasma concentrations in patients [22] (Supplementary Fig. 9a). Only KD with the most potent sh*KDM6A* #7 resulted in a significantly increased resistance to AraC (Fig. 4b). However, the effect of *KDM6A* KD on response towards DNR or 6-thioguanine (6-TG) was not as prominent or even absent: only KD cells sh*KDM6A* #7 were slightly more resistant to DNR treatment (Fig. 4c), and no change in IC_50_ values was observed after 6-TG treatment (Fig. 4d). The applied DNR concentrations (5–75 nM) are within the lower range of the reported concentrations in AML patients (403.8 ± 349 nM) [23]. Since induction therapy typically involves continuous treatment for seven (“7 + 3”) or 10 days (TAD regime), we next applied a prolonged time course with multiple treatments. Prolonged treatment with 6-TG showed no difference in the amount of viable cells between control and KD after 14 days (Fig. 4g). Differences in growth under AraC treatment started at day 4, and resulted in a significant proliferative advantage for *KDM6A* KD cells compared to control (Fig. 4e). Growth of both control groups was completely arrested under DNR treatment after day 8, whereas *KDM6A* KD cells were strongly proliferating (Fig. 4f). *KDM6A* KD efficiency and proliferative advantage under DNR were positively correlated. To compare the impact of *KDM6A* KD vs. knockout (KO), we applied CRISPR/Cas9 genome editing to ablate KDM6A expression in K562 cells. We established single cell *KDM6A* KO clones (Supplementary Fig. 9c), leading to complete loss of KDM6A expression (Fig. 4h). After 72 h AraC treatment, IC_50_ values were significantly increased for both *KDM6A* KO clones compared to controls (Fig. 4i). We observed a trend towards higher IC_50_ values or no difference between KO and control cells after DNR or 6-TG treatment, respectively (Supplementary Fig. 9d, e). These data indicate that reduction or loss of KDM6A expression in K562 cells increases resistance to AraC and DNR but not 6-TG.

### Loss of KDM6A in MM-1 recapitulates the drug phenotype of the *KDM6A* mutant sister cells MM-6

**Fig. 5:**
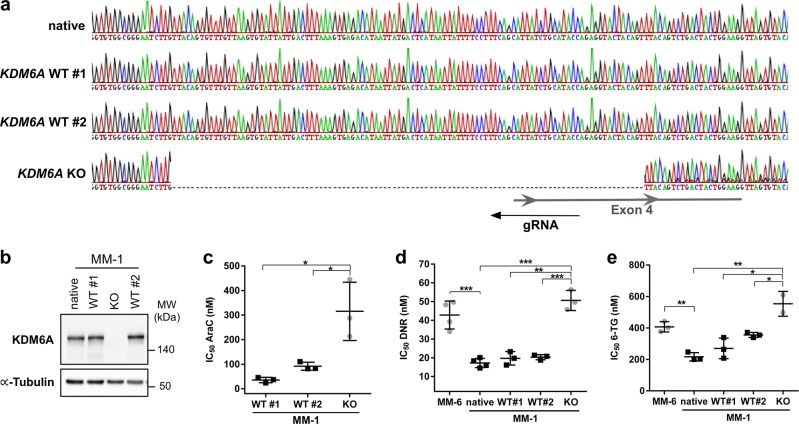
KDM6A loss in MM-1 recapitulates the drug phenotype of the *KDM6A* mutant sister cell line MM-6. **a** DNA sequencing chromatogram showing *KDM6A* frameshift mutation A112Vfs*3 of *KDM6A* knockout (KO) MM-1 clone which is absent in native MM-1 cells and two *KDM6A* WT clones. Last 74 bp of Intron 3 and 29 bp of exon 4 are deleted. WT #1 and #2 clones were tested negative for *KDM6A* KO after CRISPR/Cas9 targeting. **b** Immunoblotting for KDM6A expression in KDM6A WT and KO cells. Blot is representative of three independent experiments. MW molecular weight, α-Tubulin loading control. **c**–**e** Comparison of IC_50_ values for AraC (**c**), DNR (**d**), and 6-TG (**e**) between MM-6 and MM-1 cells including MM-1 native, two *KDM6A* WT and one *KDM6A* KO clone. Cells were treated for 96 h with the respective drug. Mean ± s.d are given for three independent experiments. Unpaired, two-tailed Student’s *t*-test; *P < 0.05; **P < 0.01; ***P < 0.001

**Fig. 6:**
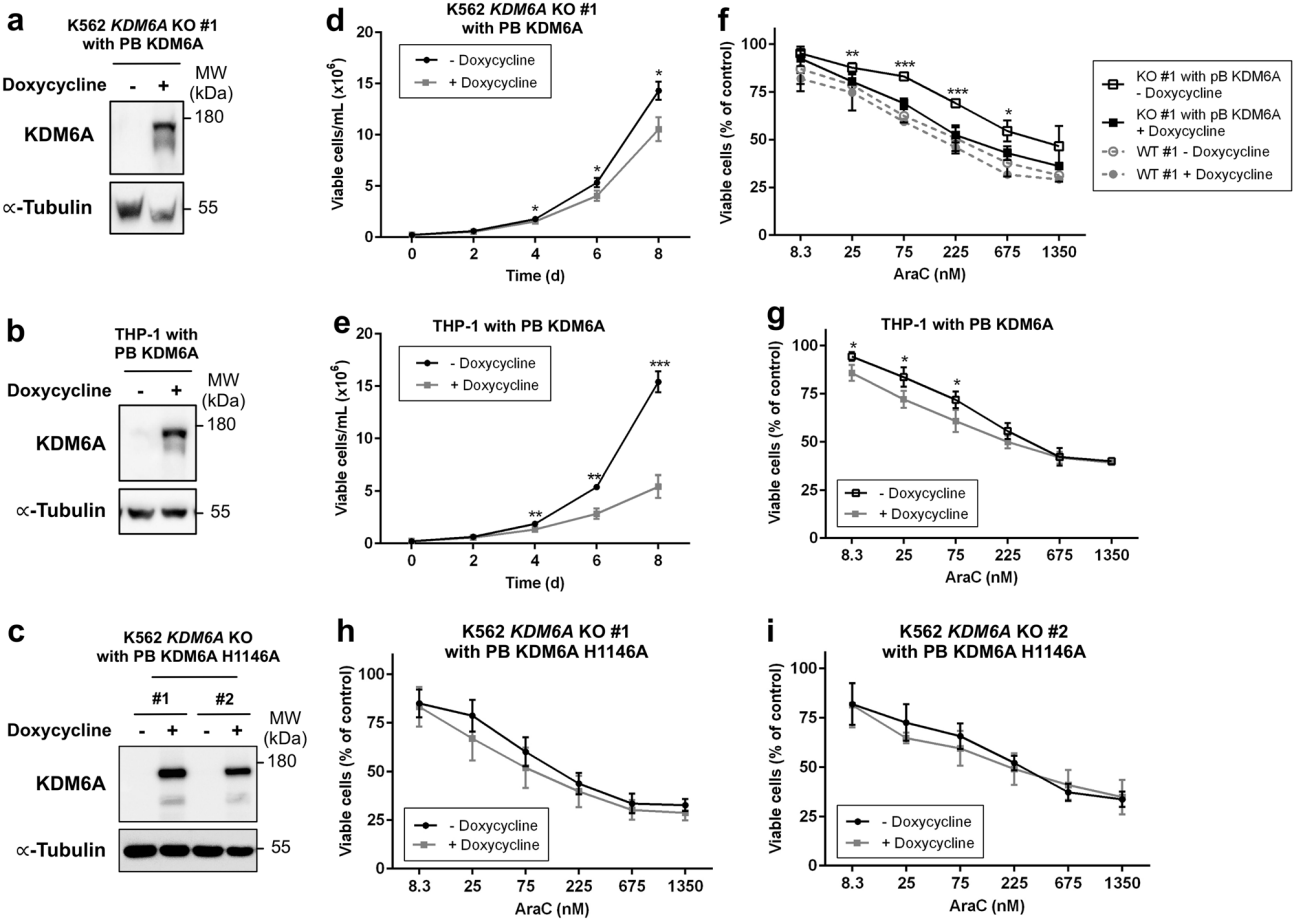
Re-expression of KDM6A suppresses cell growth and sensitizes cells to AraC therapy. **a**–**c** Immunoblot showing doxycycline inducible re-expression of KDM6A in K562 *KDM6A* KO #1 PB KDM6A (**a**), *KDM6A* mutant THP-1 PB KDM6A (**b**) and K562 *KDM6A* KO #1 and #2 PB KDM6A H1146A cells (**c**) after treatment with 0.5 μg/mL doxycycline for 48 h. Blot is representative of two independent experiments. MW molecular weight, α-Tubulin loading control. **d**, **e** Proliferation assay measuring the amount of viable K562 *KDM6A* KO #1 PB KDM6A (**d**) and *KDM6A* mutant THP-1 PB KDM6A cells (**e**) in the absence or presence of doxycycline (0.5 μg/mL) every 2 days for 8 days. Mean ± s.d. are given for three independent experiments. Unpaired, two-tailed Student’s *t*-test; *P < 0.05; **P < 0.01; ***P < 0.001. **f**–**i** AraC dose-response analysis after treatment for 72 h in K562 *KDM6A* KO #1 PB KDM6A and K562 *KDM6A* WT #1 (**f**), *KDM6A* mutant THP-1 PB KDM6A (**g**) and K562 *KDM6A* KO PB KDM6A H1146A cells (**h**,**i**) in the absence or presence of doxycycline (0.5 μg/mL). Mean ± s.d. are given for at least three independent experiments. Unpaired, two-tailed Student’s *t*-test; *P < 0.05; **P < 0.01; ***P < 0.001

The sister cell lines MM-1 and MM-6 have originally been established in culture from the same male AML patient at relapse [24]. Whereas MM-1 cells express KDM6A, MM-6 cells harbor a *KDM6A* exon deletion, rendering them a good model to examine the implications of KDM6A loss within a similar genetic background. To investigate whether *KDM6A* KO in MM-1 cells results in the same drug resistance phenotype observed in MM-6 cells (Fig. 5d, e), we applied CRISPR/Cas9 targeting the *KDM6A* locus in MM-1 cells (Fig. 5a). Compared to MM-1 parental and WT single cell clones, KDM6A expression was lost in one clone (Fig. 5b). In agreement with our observation that MM-6 cells are 4.3-fold more resistant to AraC after 72 h than MM-1 (Supplementary Fig. 7a), *KDM6A* KO in MM-1 increased the AraC IC_50_ compared to both WT clones (3.4 to 8.8-fold increase after 96 h treatment; Fig. 5c). Comparison of the IC_50_ values after DNR (Figs. 5d) and 6-TG (Fig. 5e) treatment indicated that *KDM6A* KO MM-1 are significantly less sensitive to DNR and 6-TG than *KDM6A* WT MM-1 cells. Strikingly, KO of *KDM6A* in MM-1 conferred a similar resistance to DNR (MM-1: KO vs. WT#1/#2: 2.5 to 2.6-fold; native MM-1 vs. MM-6: 2.5-fold increase in IC_50_) and 6-TG (MM-1: KO vs. WT#1/#2: 1.6 to 2.1-fold; native MM-1 vs. MM-6: 1.9-fold) as in MM-6 cells. Together these data suggest that loss of KDM6A in MM-1 is responsible for a decreased sensitivity towards AraC, DNR, and 6-TG.

### Re-expression of KDM6A sensitizes cells to AraC treatment

To investigate if re-introduction of KDM6A in *KDM6A*-null cell lines sensitizes them to AraC treatment, we generated stable cell lines with a doxycycline inducible PiggyBac (PB) KDM6A expression system (Fig. 6a–c). Re-expression of KDM6A significantly suppressed proliferation in THP-1 and K562 *KDM6A* KO cells (Fig. 6d, e). Furthermore, re-expression of KDM6A significantly decreased the amount of viable cells after treatment with AraC in both cell lines (Fig. 6f, g). *KDM6A* KO K562 cells, which were more resistant to AraC than *KDM6A* WT cells, were sensitized again to AraC treatment by re-expressing KDM6A (Fig. 6f). To investigate if the demethylase activity of KDM6A is essential for re-sensitizing cells to AraC treatment, we expressed a catalytically dead *KDM6A* mutant, H1146A [25], in the K562 *KDM6A* KO #1 and #2 cells. Expression of KDM6A H1146A showed only a trend in decreasing the amount of viable cells after treatment with AraC (Fig. 6h, i).

### Decreased ENT1 expression by *KDM6A* loss mediates increased AraC resistance

**Fig. 7:**
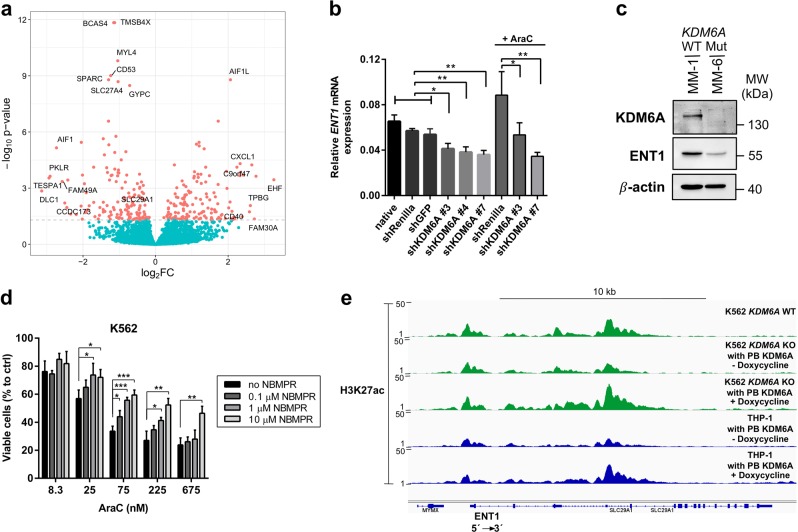
Downregulation of ENT1 by KDM6A loss is regulated by H3K27 acetylation and increases AraC resistance. **a** Volcano plot showing log_2_ fold change on the x-axis and adjusted *P* value on the y-axis for the differential gene expression between shRNA-mediated KD of *KDM6A* (sh*KDM6A* #7) and control (sh*GFP*) in K562 cells (*n* = 6). Genes with adjusted *P* value < 0.05 are highlighted in red and those with a log_2_ FC >2.5 or <−2.5 are labeled with the gene name. In addition, genes with adjusted *P* value < 1e-8 and the gene *SLC29A1* are labeled. **b** qRT-PCR for *ENT1* in K562 cells: native, sh*Renilla*, sh*GFP* and three different sh*KDM6A* KDs. *ENT1* mRNA for sh*Renilla*, sh*KDM6A* #3 and sh*KDM6A* #7 K562 cells is also shown after treatment with 150 nM AraC for 72 h. The mean ± s.d. for *ENT1* mRNA relative to *GAPDH* for three independent experiments is shown. **c** Immunoblotting showing strong reduction of ENT1 protein in *KDM6A* mutant MM-6 cells compared to the *KDM6A* WT sister cell line MM-1. Immunoblots are representative of three independent experiments. MW, molecular weight; β-actin, loading control. **d** Inhibition of ENT1 by NBMPR increases the amount of viable cells during AraC treatment. K562 cells were treated with different AraC concentrations in combination with 0, 0.1, 1 and 10 μM of NBMPR for 72 h. Mean ± s.d are given for three independent experiments. Unpaired, two-tailed Student’s *t*-test; *P < 0.05; **P < 0.01; ***P < 0.001. **e** Genomic snapshot of H3K27ac ChIP-seq in K562 *KDM6A* WT, K562 *KDM6A* KO PB KDM6A and *KDM6A* mutant THP-1 cells in the absence or presence of doxycycline at the *ENT1* locus. Cells were treated with media +/− doxycycline (0.5 μg/mL) every 24 h for 72 h

To identify genes involved in KDM6A-mediated drug resistance, we performed RNA-Seq analysis in K562 cells treated with siRNA (Supplementary Fig. 10a, b) or shRNA against *KDM6A* (Fig. 4a). Transient KDM6A KD with siRNA, which showed a trend towards higher IC_50_ for AraC compared to control (Supplementary Fig. 10c), resulted in transcriptional downregulation of 39 genes and upregulation of 7 genes (Supplementary Fig. 10d). For the most potent sh*KDM6A* #7 we detected transcriptional deregulation of 295 genes compared to 7 or 54 deregulated genes during sh*KDM6A* #3 or #4 mediated KD, respectively (Supplementary Fig. 10e). Whereas the majority of differentially expressed genes (39/46) was downregulated in the siRNA-mediated KD (Supplementary Fig. 10d), sh*KDM6A* #7 KD resulted in similar transcriptional down- (150, 50.8%) and upregulation (145, 49.2%; Fig. 7a). Treatment with AraC (150 nM) during shRNA-mediated KD led to increased transcriptional deregulation (sh*KDM6A* #7: 2,357; sh*Renilla*: 2,272) in comparison to the native state with 40.3% of genes exclusively being deregulated in sh*KDM6A* #7 (Supplementary Fig. 10f). Next, we compared KDM6A regulated genes with known candidate genes in drug metabolic pathways and found that *ENT1* was consistently downregulated in KDM6A KD cells in both RNA-Seq screenings (Fig. 7a, Supplementary Fig. 10d). ENT1, or SLC29A1, is a membrane transporter important for the cellular uptake of nucleosides and its analogues [26]. Sh*KDM6A* K562 cells showed significantly reduced *ENT1* mRNA compared to controls (Fig. 7b). AraC treatment slightly increased *ENT1* mRNA expression in sh*Renilla* cells. In sh*KDM6A* #7 cells *ENT1* expression was reduced even after AraC administration (Fig. 7b). Additionally, decreased ENT1 expression was detected in *KDM6A* KO K562 (Supplementary Fig. 11a) and MM-6 cells (Fig. 7c). Treatment of K562, MM-1, and MM-6 cells with a selective ENT1 inhibitor, NBMPR, in combination with AraC resulted in increased cell survival compared to AraC alone (Fig. 7d, Supplementary Fig. 11b, c). By contrast, no change was observed when we combined ENT1 inhibition with DNR or 6-TG in K562 cells (Supplementary Fig. 11d, e). To further elucidate the mechanism of ENT1 regulation by KDM6A, we performed ChIP-seq analysis for H3K27me3 and H3K27ac in MM-1 and MM-6 cells as recent studies have reported that the tumor suppressor effect is largely demethylase independent [19, 27]. ChIP-seq for H3K27me3 showed no enrichment on the *ENT1* locus, however, we detected differential H3K27ac peaks in the promoter and a putative enhancer region of ENT1 in MM-1 compared to MM-6 (Supplementary Fig. 12). Additionally, K562 and THP-1 cells with loss of KDM6A showed low H3K27ac peaks on the *ENT1* locus, which were increased upon doxycycline inducible re-expression of KDM6A in both cell lines (Fig. 7e). In summary, our data demonstrate that strong reduction or complete loss of KDM6A decreases ENT1 expression, probably through direct or indirect effects of KDM6A on enhancer regions, which then promotes increased AraC resistance.

## Discussion

In cancer, except for bladder cancer [28], the frequency of mutation in the *KDM6A* gene is rather low [29, 30]. In AML, frequency ranges from 0.7% [31] to 4.0% [3, 32, 33] and the majority of mutations are missense mutations. In our study, *KDM6A* mutations were exclusively missense and truncating mutations. One of these mutations, E1325X, has been previously described in an AML patient at diagnosis and was present in a subclone only [31]. Reduced expression or mutations/deletions of KDM6A correlate with poor overall survival in patients with CN-AML [3] or myeloma [29], respectively. We identified three AML patients harboring *KDM6A* mutations at diagnosis and observed an outgrowth of the *KDM6A* mutated population at relapse. These results are in agreement with our previous study [3] and suggest that *KDM6A* loss may contribute to increased chemo-resistance in AML.

As *KDM6A* is not X-inactivated [34], females with T-ALL benefit from two functional copies [17], and compared to female patients shortened relapse-free survival is seen in male CN-AML patients [3]. Dependent on expression dosage, KDM6A deficiency was found to accelerate and promote cancer progression in a mouse lymphoma model [35]. We observed that KDM6A mRNA and protein expression is reduced in 45.7% and 44.4% of AML patients at relapse, respectively. As most of these samples showed no *KDM6A* mutation, another mechanism contributing to KDM6A regulation might be DNA methylation. We found that AML patients with high DNA methylation levels of KDM6A have a significantly shorter overall survival. These results are in line with our previous study [3] showing a correlation of low KDM6A expression and poor overall survival in CN-AML patients.

In agreement with our data showing higher AraC resistance in *KDM6A* mutant AML cell lines, we observed that a *KDM6A* mutant PDX sample is less sensitive towards in vitro AraC treatment compared to a *KDM6A* WT PDX sample. Prominent differences in treatment response during in vivo therapy indicate that even small differences in drug sensitivity observed in vitro can significantly impact long-term in vivo therapy.

We have recently demonstrated that *KDM6A* mutant MM-6 are less sensitive to AraC than MM-1 cells [3]. Under competitive growth conditions, we observed a selective growth advantage of MM-6 during AraC treatment. Furthermore, inducible re-expression of KDM6A in *KDM6A*-null cells sensitized to AraC treatment. Very recently, Gozdecka et al. [19] could show that lentiviral expression of KDM6A in MM-6 cells suppressed spontaneous cell proliferation. The data presented here extend these findings and show that KDM6A acts as a tumor suppressor and mediates drug resistance in AML.

UTY, a catalytically inactive KDM6A homolog that is encoded on the Y chromosome, was recently shown to suppress myeloid leukemogenesis in *KDM6A*-deficient male mice [19]. It was also reported to be lost or reduced in conjunction with *KDM6A* mutations in pancreatic cancers with squamous differentiation in male patients [27]. We found that UTY mRNA expression was lost or barely detectable in MM-6 and MM-1 cells suggesting that the drug resistant phenotype of MM-6 is not affected by UTY.

Various mechanisms of drug resistance in AML have been proposed in the last decades. AraC for instance can only exert its cytotoxic effect after cellular uptake and conversion into its active form. Among the key candidate genes in AraC metabolism, we consistently found differential expression of the drug influx transporter gene *ENT1*. We demonstrate that KD/KO of *KDM6A* leads to decreased expression of ENT1 linking decreased KDM6A levels to increased AraC resistance. Previous studies showed that KD or inhibition of ENT1 in AML cell lines confers AraC resistance [36, 37]. In AraC based therapy, AML patients with low ENT1 levels were reported to have shorter disease-free or overall survival [38]. In agreement with previous results, inhibition of ENT1 by NBMPR increased AraC resistance. Furthermore, we demonstrate that transport of DNR and 6-TG across the cell membrane is ENT1 independent. Recently, KDM6A was reported to regulate gene expression during myeloid leukemogenesis mainly by modifying levels of H3K27 acetylation, H3K4 monomethylation and chromatin accessibility [19]. Our ChIP-seq analysis suggests that ENT1 expression is regulated by H3K27 acetylation whereas H3K27 demethylase activity is dispensable for ENT1 expression. As re-expression of a catalytically dead mutant in K562 *KDM6A* KO cells had not the same effect as *KDM6A* WT in re-sensitizing cells to AraC, it remains to be determined if disruption of the catalytic domain impacts the regulation of ENT1 expression.

Prolonging the treatment time for DNR, resulted in a significant increase in DNR resistance in MM-6 cells compared to a shorter treatment as previously described [3]. Additionally, we demonstrate that deletion of *KDM6A* in MM-1 recapitulates the same drug resistant phenotype observed in MM-6. The mechanisms leading to DNR resistance need further investigation, but loss of KDM6A-mediated upregulation of the DNR metabolizing enzymes AKR1C1 and AKR1C2 (data not shown) might be involved in DNR resistance. A study demonstrated that upregulation of AKR1C1/3 facilitated reduction of DNR efficacy in leukemic U937 cells [39].

Taken together, our results show that KDM6A inactivation either by loss-of-function mutations or protein downregulation mediates drug resistance in AML.

## Supplementary information

Supplementary Table 1

Supplementary Table 2

Supplementary Figures and Material

## References

[1] Döhner H, Estey E, Grimwade D, Amadori S, Appelbaum FR, Büchner T (2017). Diagnosis and management of AML in adults: 2017 ELN recommendations from an international expert panel. Blood.

[2] Döhner H, Weisdorf DJ, Bloomfield CD (2015). Acute Myeloid Leukemia. N Engl J Med.

[3] Greif PA, Hartmann L, Vosberg S, Stief SM, Mattes R, Hellmann I (2018). Evolution of cytogenetically normal acute myeloid leukemia during therapy and relapse: an exome sequencing study of 50 patients. Clin Cancer Res.

[4] Hong S, Cho Y-W, Yu L-R, Yu H, Veenstra TD, Ge K (2007). Identification of JmjC domain-containing UTX and JMJD3 as histone H3 lysine 27 demethylases. Proc Natl Acad Sci USA.

[5] Agger K, Cloos PAC, Christensen J, Pasini D, Rose S, Rappsilber J (2007). UTX and JMJD3 are histone H3K27 demethylases involved in HOX gene regulation and development. Nature.

[6] Min GL, Villa R, Trojer P, Norman J, Yan KP, Reinberg D (2007). Demethylation of H3K27 regulates polycomb recruitment and H2A ubiquitination. Science.

[7] Hu D, Gao X, Morgan MA, Herz H-M, Smith ER, Shilatifard A (2013). The MLL3/MLL4 branches of the COMPASS family function as major histone H3K4 monomethylases at enhancers. Mol Cell Biol.

[8] Dhar SS, Zhao D, Lin T, Gu B, Pal K, Wu SJ (2018). MLL4 is required to maintain broad H3K4me3 peaks and super-enhancers at tumor suppressor genes. Mol Cell.

[9] Froimchuk E, Jang Y, Ge K (2017). Histone H3 lysine 4 methyltransferase KMT2D. Gene.

[10] Pasini D, Malatesta M, Jung HR, Walfridsson J, Willer A, Olsson L (2010). Characterization of an antagonistic switch between histone H3 lysine 27 methylation and acetylation in the transcriptional regulation of Polycomb group target genes. Nucleic Acids Res.

[11] Creyghton MP, Cheng AW, Welstead GG, Kooistra T, Carey BW, Steine EJ (2010). Histone H3K27ac separates active from poised enhancers and predicts developmental state. Proc Natl Acad Sci USA.

[12] van Haaften G, Dalgliesh GL, Davies H, Chen L, Bignell G, Greenman C (2009). Somatic mutations of the histone H3K27 demethylase gene UTX in human cancer. Nat Genet.

[13] Ross JS, Badve S, Wang K, Sheehan CE, Boguniewicz AB, Otto GA (2015). Genomic profiling of advanced-stage, metaplastic breast carcinoma by next-generation sequencing reveals frequent, targetable genomic abnormalities and potential new treatment options. Arch Pathol Lab Med.

[14] Nickerson ML, Dancik GM, Im KM, Edwards MG, Turan S, Brown J (2014). Concurrent alterations in TERT, KDM6A, and the BRCA pathway in bladder cancer. Clin Cancer Res.

[15] Huether R, Dong L, Chen X, Wu G, Parker M, Wei L (2014). The landscape of somatic mutations in epigenetic regulators across 1,000 paediatric cancer genomes. Nat Commun.

[16] Ntziachristos P, Tsirigos A, Welstead GG, Trimarchi T, Bakogianni S, Xu L (2014). Contrasting roles of histone 3 lysine 27 demethylases in acute lymphoblastic leukaemia. Nature.

[17] Van der Meulen J, Sanghvi V, Mavrakis K, Durinck K, Fang F, Matthijssens F (2015). The H3K27me3 demethylase UTX is a gender-specific tumor suppressor in T-cell acute lymphoblastic leukemia. Blood.

[18] Bolli N, Manes N, McKerrell T, Chi J, Park N, Gundem G (2015). Characterization of gene mutations and copy number changes in acute myeloid leukemia using a rapid target enrichment protocol. Haematologica.

[19] Gozdecka Malgorzata, Meduri Eshwar, Mazan Milena, Tzelepis Konstantinos, Dudek Monika, Knights Andrew J., Pardo Mercedes, Yu Lu, Choudhary Jyoti S., Metzakopian Emmanouil, Iyer Vivek, Yun Haiyang, Park Naomi, Varela Ignacio, Bautista Ruben, Collord Grace, Dovey Oliver, Garyfallos Dimitrios A., De Braekeleer Etienne, Kondo Saki, Cooper Jonathan, Göttgens Berthold, Bullinger Lars, Northcott Paul A., Adams David, Vassiliou George S., Huntly Brian J. P. (2018). UTX-mediated enhancer and chromatin remodeling suppresses myeloid leukemogenesis through noncatalytic inverse regulation of ETS and GATA programs. Nature Genetics.

[20] Vick B, Rothenberg M, Sandhöfer N, Carlet M, Finkenzeller C, Krupka C (2015). An advanced preclinical mouse model for acute myeloid leukemia using patients’ cells of various genetic subgroups and in vivo bioluminescence imaging. PLoS ONE.

[21] Figueroa ME, Lugthart S, Li Y, Erpelinck-Verschueren C, Deng X, Christos PJ (2010). DNA methylation signatures identify biologically distinct subtypes in acute myeloid leukemia. Cancer Cell.

[22] Early AP, Preisler HD, Slocum H, Rustum YM (1982). A pilot study of high-dose 1-beta-D-arabinofuranosylcytosine for acute leukemia and refractory lymphoma: clinical response and pharmacology. Cancer Res.

[23] Bogason A, Quartino AL, Lafolie P, Masquelier M, Karlsson MO, Paul C (2011). Inverse relationship between leukaemic cell burden and plasma concentrations of daunorubicin in patients with acute myeloid leukaemia. Br J Clin Pharmacol.

[24] MacLeod RA, Voges M, Drexler HG (1993). Mono Mac 6: a mature monoblastic leukemia cell line with t(9;11)(p21; q23). Blood.

[25] Wang C, Lee J-E, Cho Y-W, Xiao Y, Jin Q, Liu C (2012). UTX regulates mesoderm differentiation of embryonic stem cells independent of H3K27 demethylase activity. Proc Natl Acad Sci USA.

[26] M Pastor-Anglada, S Perez-Torras, Nucleoside transporter proteins as biomarkers of drug responsiveness and drug targets, Front. Pharmacol. 2015; 6. 10.3389/fphar.2015.00013. 10.3389/fphar.2015.00013 PMC4322540 25713533

[27] Andricovich J, Perkail S, Kai Y, Casasanta N, Peng W, Tzatsos A (2018). Loss of KDM6A activates super-enhancers to induce gender-specific squamous-like pancreatic cancer and confers sensitivity to BET inhibitors. Cancer Cell.

[28] Ler LD, Ghosh S, Chai X, Thike AA, Heng HL, Siew EY (2017). Loss of tumor suppressor KDM6A amplifies PRC2-regulated transcriptional repression in bladder cancer and can be targeted through inhibition of EZH2. Sci Transl Med.

[29] Pawlyn C, Kaiser MF, Heuck C, Melchor L, Wardell CP, Murison A (2016). The spectrum and clinical impact of epigenetic modifier mutations in Myeloma. Clin Cancer Res.

[30] J-F Spinella, P Cassart, C Richer, V Saillour, M Ouimet, S Langlois, et al. Genomic characterization of pediatric T-cell acute lymphoblastic leukemia reveals novel recurrent driver mutations, Oncotarget. 2016;7. 10.18632/oncotarget.11796. 10.18632/oncotarget.11796 PMC5323170 27602765

[31] Papaemmanuil E, Gerstung M, Bullinger L, Gaidzik VI, Paschka P, Roberts ND (2016). Genomic Classification and Prognosis in Acute Myeloid Leukemia. N Engl J Med.

[32] Metzeler KH, Herold T, Rothenberg-Thurley M, Amler S, Sauerland MC, Dennis G (2016). Spectrum and prognostic relevance of driver gene mutations in acute myeloid leukemia. Blood.

[33] The Cancer Genome Atlas Research Network. (2013). Genomic and epigenomic landscapes of adult de novo acute myeloid leukemia. N Engl J Med.

[34] Greenfield A, Carrel L, Pennisi D, Philippe C, Quaderi N, Siggers P (1998). The UTX gene escapes X inactivation in mice and humans. Hum Mol Genet.

[35] Li X, Zhang Y, Zheng L, Liu M, Chen CD, Jiang H (2018). UTX is an escape from X-inactivation tumor-suppressor in B cell lymphoma. Nat Commun.

[36] Kim JH, Lee C, Cheong HS, Koh Y, Ahn KS, Kim HL (2016). SLC29A1 (ENT1) polymorphisms and outcome of complete remission in acute myeloid leukemia. Cancer Chemother Pharmacol.

[37] Macanas-Pirard P, Broekhuizen R, González A, Oyanadel C, Ernst D, García P (2017). Resistance of leukemia cells to cytarabine chemotherapy is mediated by bone marrow stroma, involves cell-surface equilibrative nucleoside transporter-1 removal and correlates with patient outcome. Oncotarget.

[38] Wan H, Zhu J, Chen F, Xiao F, Huang H, Han X (2014). SLC29A1 single nucleotide polymorphisms as independent prognostic predictors for survival of patients with acute myeloid leukemia: an in vitro study. J Exp Clin Cancer Res.

[39] Matsunaga T, Yamaguchi A, Morikawa Y, Kezuka C, Takazawa H, Endo S (2014). Induction of aldo-keto reductases (AKR1C1 and AKR1C3) abolishes the efficacy of daunorubicin chemotherapy for leukemic U937 cells. Anticancer Drugs.

[40] Liu W, Xie Y, Ma J, Luo X, Nie P, Zuo Z (2015). IBS: an illustrator for the presentation and visualization of biological sequences. Bioinformatics.

