# Supplementary material for: Loss of KDM6A confers drug resistance in acute myeloid leukemia
Source: Leukemia. 2019 Jun 14;34(1):50–62. doi: 10.1038/s41375-019-0497-6 (PMC7214274; doi:10.1038/s41375-019-0497-6)
Supplement: Supplementary file 3 — Supplementary Figures and Material [file 41375_2019_497_MOESM3_ESM.pdf]

Supplemental Figures, Table and Methods

Loss of KDM6A confers drug resistance in acute myeloid leukemia Stief *et al*.

Table of Contents

Supplementary Figures 2

Supplementary Table 1 14

Supplementary Methods 15

Supplementary References 20

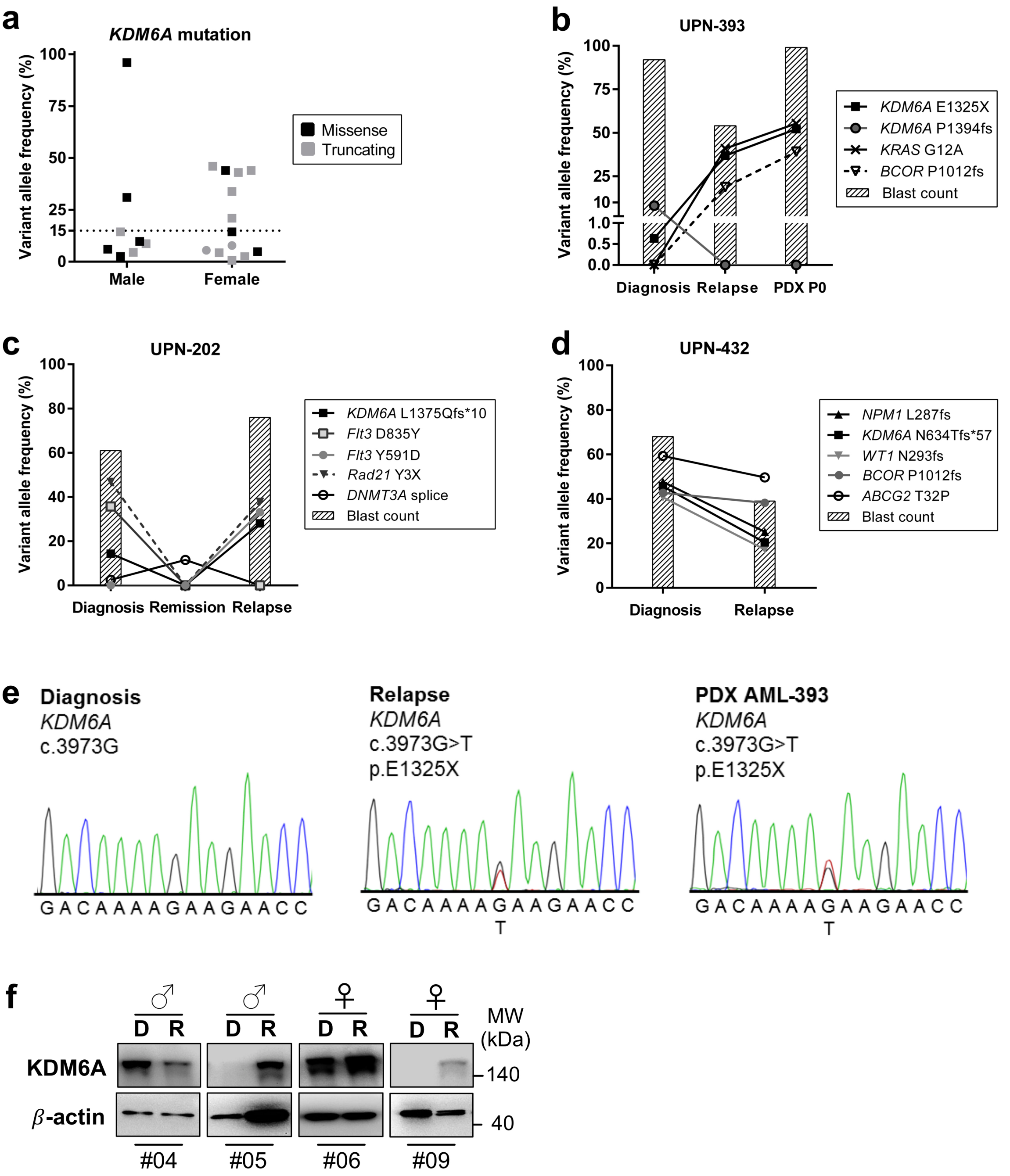

Supplementary Fig. 1, related to Fig. 1. Outgrowth of *KDM6A* mutations at relapse. a, Overview of variant allele frequency (VAF) of *KDM6A* mutations at diagnosis in 20 AML patients. The types of mutation including missense and truncating mutations are highlighted with their respective color. VAFs are shown separately for male and female patients and a VAF of 15% is pointed out by a dotted line. Presented *KDM6A* mutations are from AMLCG-99 trial (NCT00266136), AMLCG-2008 trial (NCT01382147), a CN-AML diagnosis-relapse cohort[1] and this work. One female patient harboring two *KDM6A* mutations is shown with a circle. **b,c,d,** VAF plots for different evolutionary patterns observed from diagnosis to relapse in three AML patients. Bars represent the blast count and each line represents one mutation. In addition, for patient UPN-393 (**b**) and UPN-202 (**c**), VAFs of a PDX sample from passage 0 (P0) or a remission sample are shown, respectively. **e,** DNA sequencing chromatogram showing a *KDM6A* mutation in the

gDNA of a female primary AML patient sample at relapse and in PDX AML-393 cells, established from the primary relapse sample. The mutation is not detectable in diagnosis material of the same patient. **f**, Immunoblotting for KDM6A protein expression in 4 AML patients at diagnosis (D) and relapse (R). Their respective gender is shown on top. The ratio of KDM6A to β-actin expression is shown in Figure 1d. MW, molecular weight; β-actin, loading control.

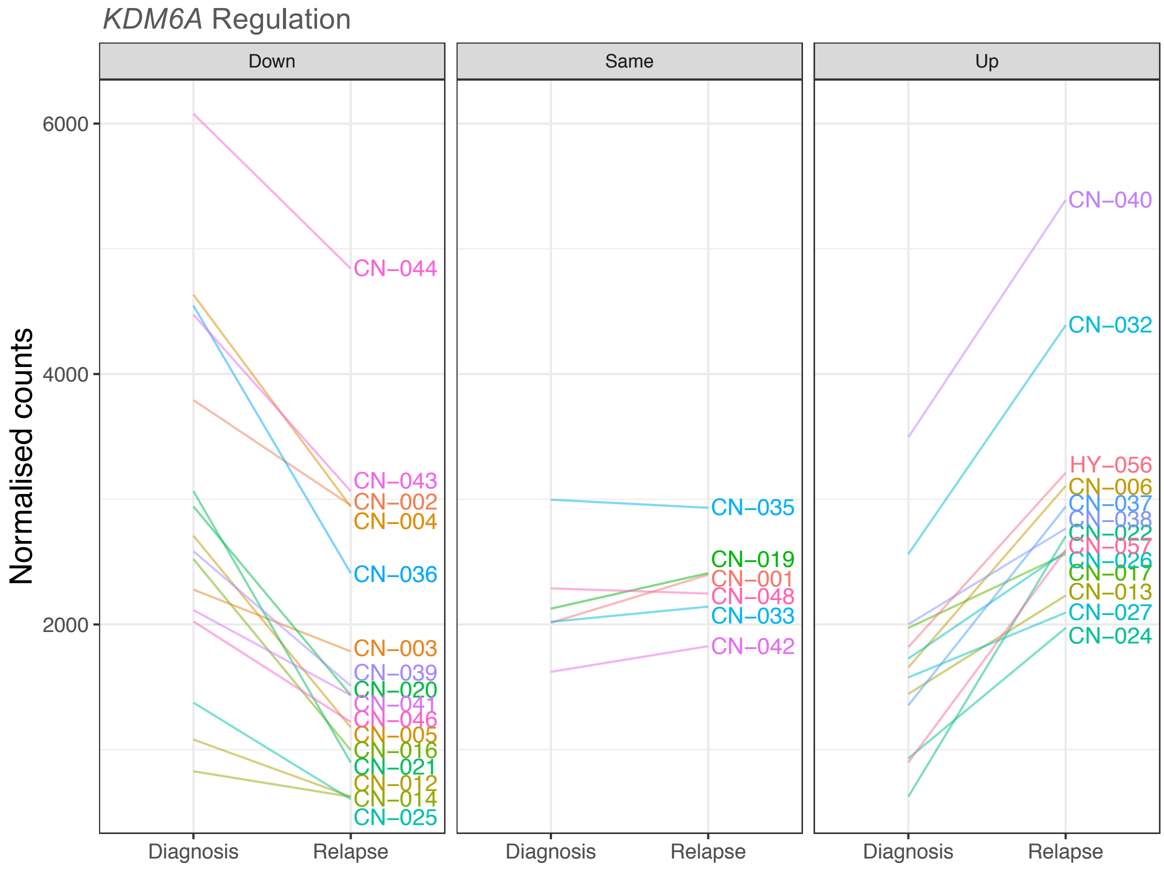

Supplementary Fig. 2, related to Fig. 1. Regulation of *KDM6A* mRNA expression in 35 CN-AML patients. The three groups, *KDM6A*-up, *KDM6A*-down and *KDM6A*-same (no change) were defined as a change in expression between diagnosis and relapse of above or below 20% respectively. Regulation of *KDM6A* was determined based on 35 previously described CN-AML patients[1].

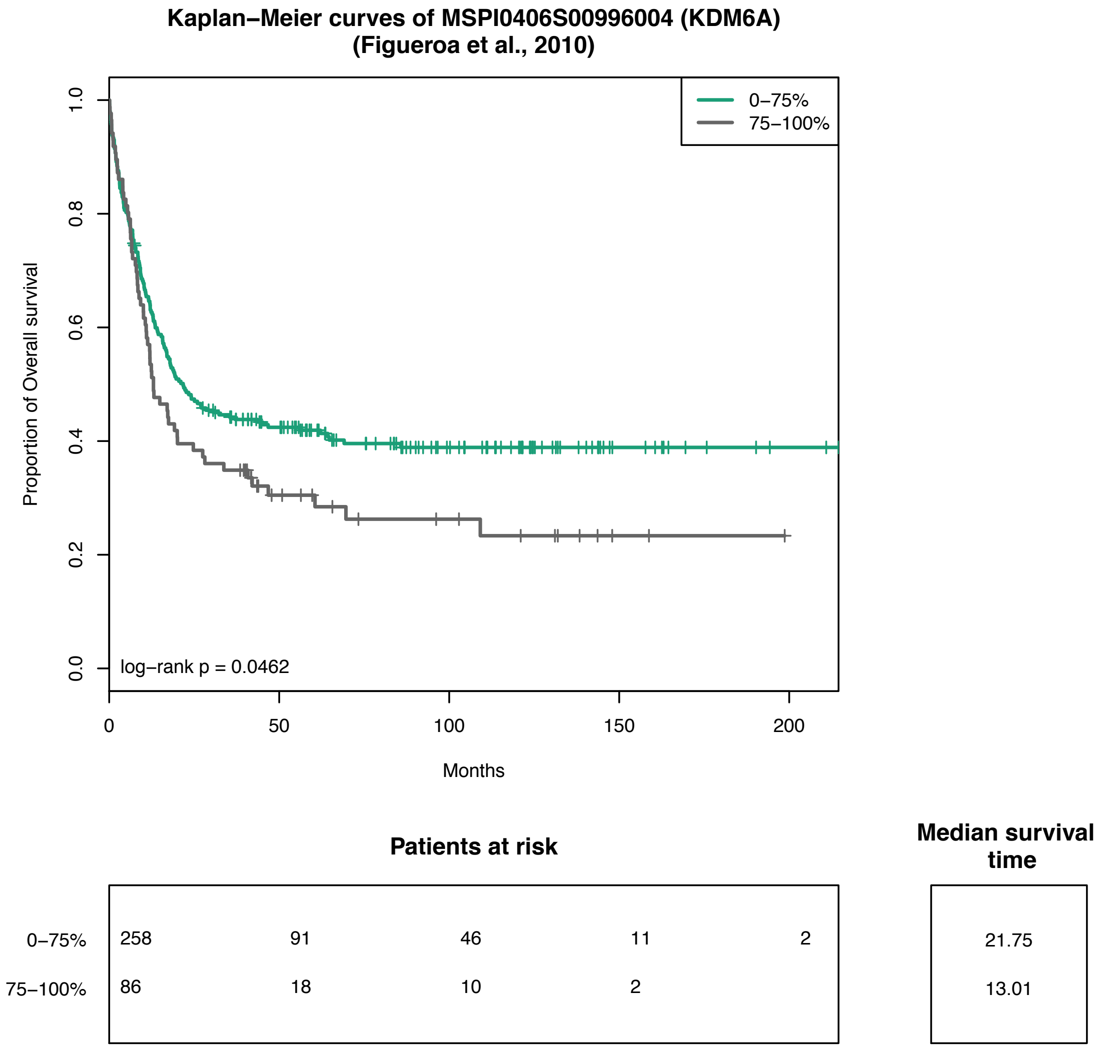

Supplementary Fig. 3, related to Fig. 1. AML patients with high DNA methylation levels of KDM6A show a shorter overall survival. The clinical correlation of KDM6A methylation status in 344 previously described AML patients[2] (top 25% versus 0-75%) and overall survival is shown.

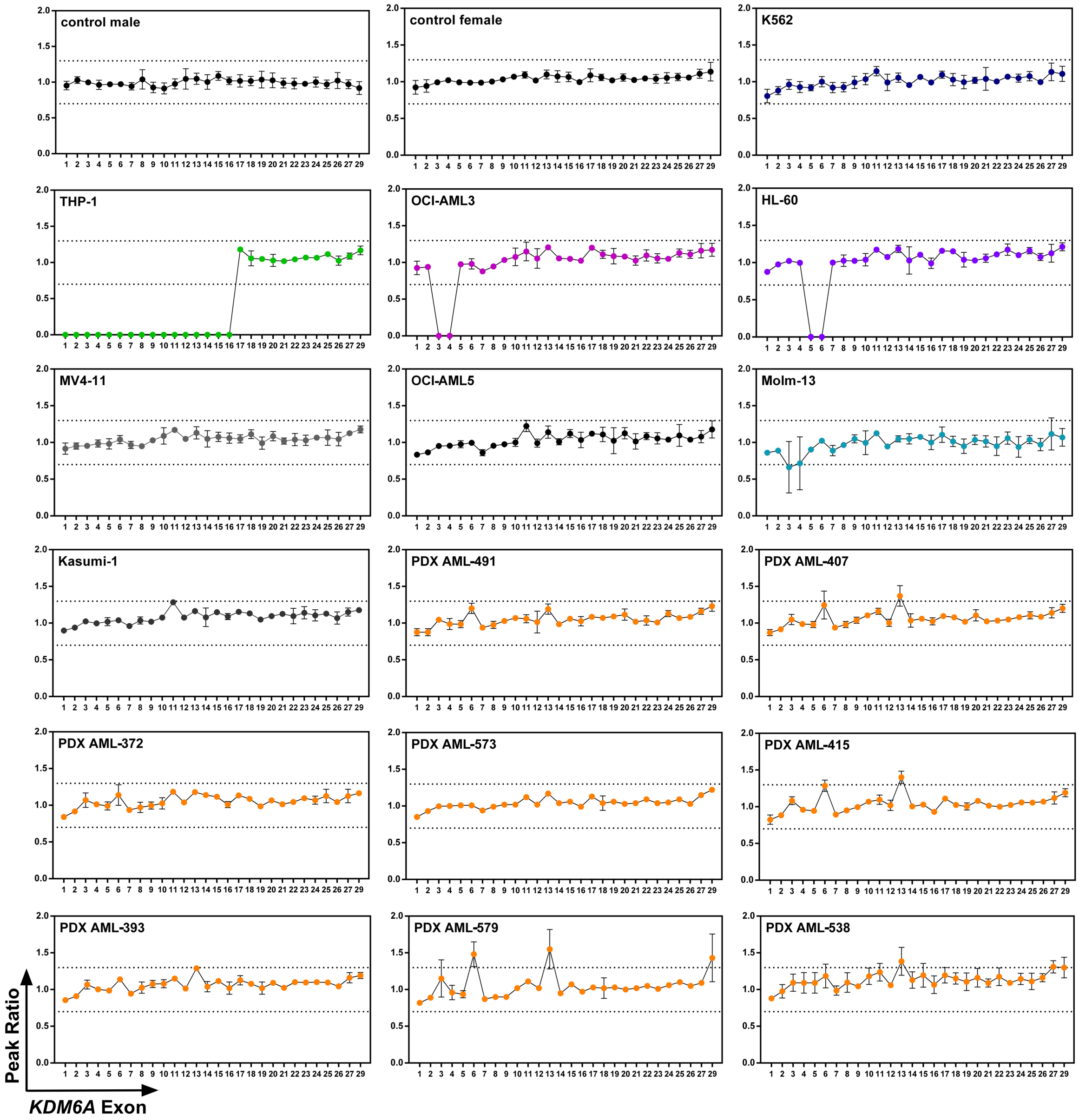

Supplementary Fig. 4, related to Fig. 2 and Fig. 3. *KDM6A* exon deletions in AML cell lines and AML PDX samples identified. The peak ratio for each *KDM6A* exon specific probe, detected by quantitative MLPA analysis, is shown. Results for 8/40 of the investigated myeloid cell lines (summarized in **Supplementary Table 1**) and 8/8 PDX AML cells are shown. The area of a normal peak ratio lies within the two dotted lines and ranges from 0.7 to 1.3. Mean ± s.d. are given for at least two independent experiments.

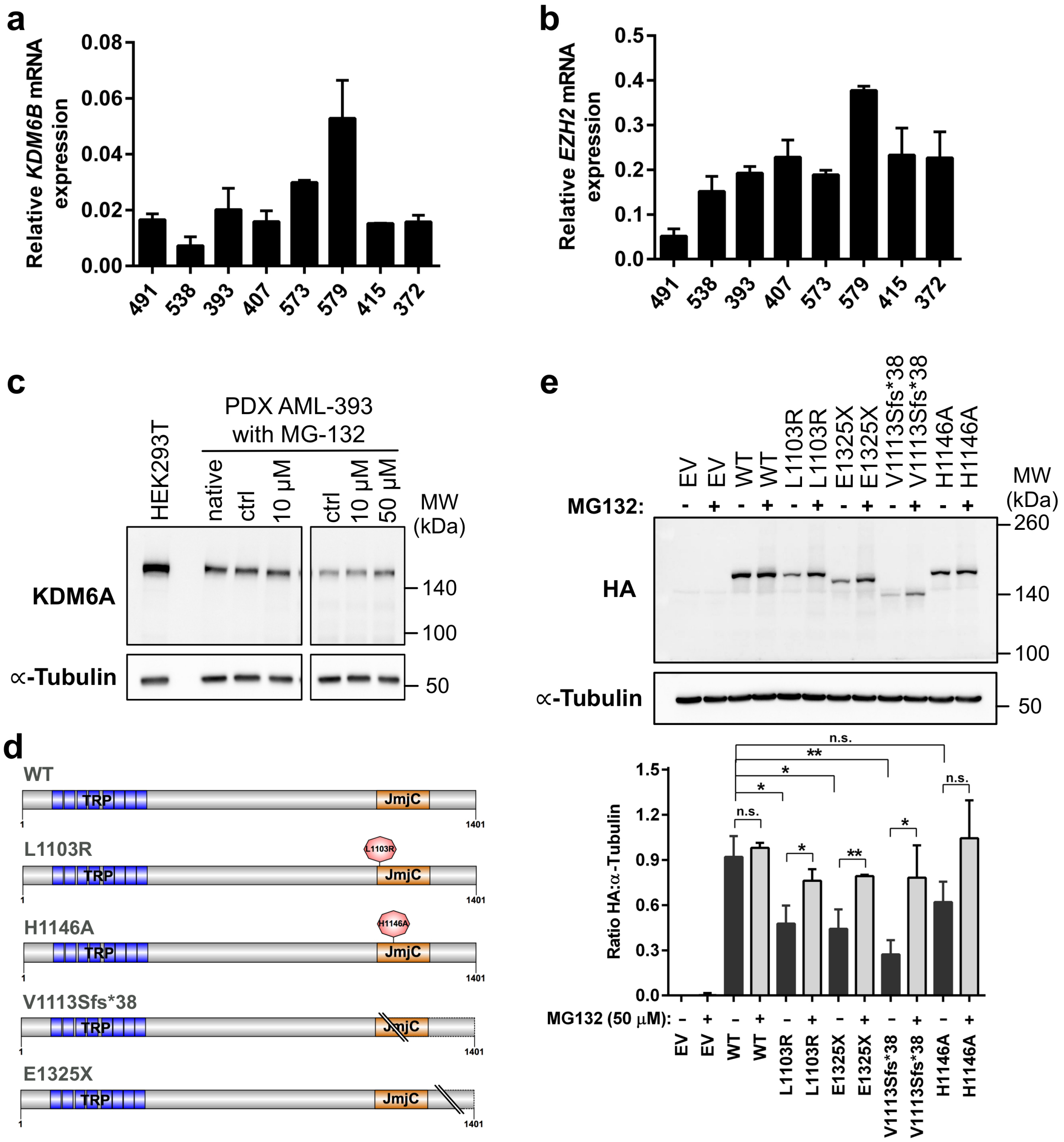

Supplementary Fig. 5, related to Fig. 2. *KDM6A* mutations are prone to degradation. a,b, qRT-PCR for *KDM6B* (**a**) and *EZH2* (**b**) in PDX relapsed AML cells. The mean ± s.d. relative to the endogenous control *GAPDH* for three experiments is shown. **c,** Immunoblot showing KDM6A expression in PDX AML-393 cells after treatment with the proteasomal inhibitor MG-132 for 6 hours. MW, molecular weight; α-Tubulin, loading control. **d,** Schematic overview of *KDM6A* WT and mutant structures illustrated with IBS software[3]. Point mutations are highlighted with a red dot. TRP, tetratricopeptide repeat; JmjC, Jumonji C. **e,** Effect of MG132 treatment (50 µM, 6h) on KDM6A expression in HEK293T cells transfected with KDM6A WT or mutants (N-terminal HA tag) is shown. Immunoblots are representative of three independent experiments. The mean ratio of HA: α-Tubulin expression ± s.d is given for three independent experiments. Unpaired, two-tailed Student's *t*-test; *P<0.05; **P<0.01; n.s., not significant. EV, empty vector; MW, molecular weight; α-Tubulin, loading control.

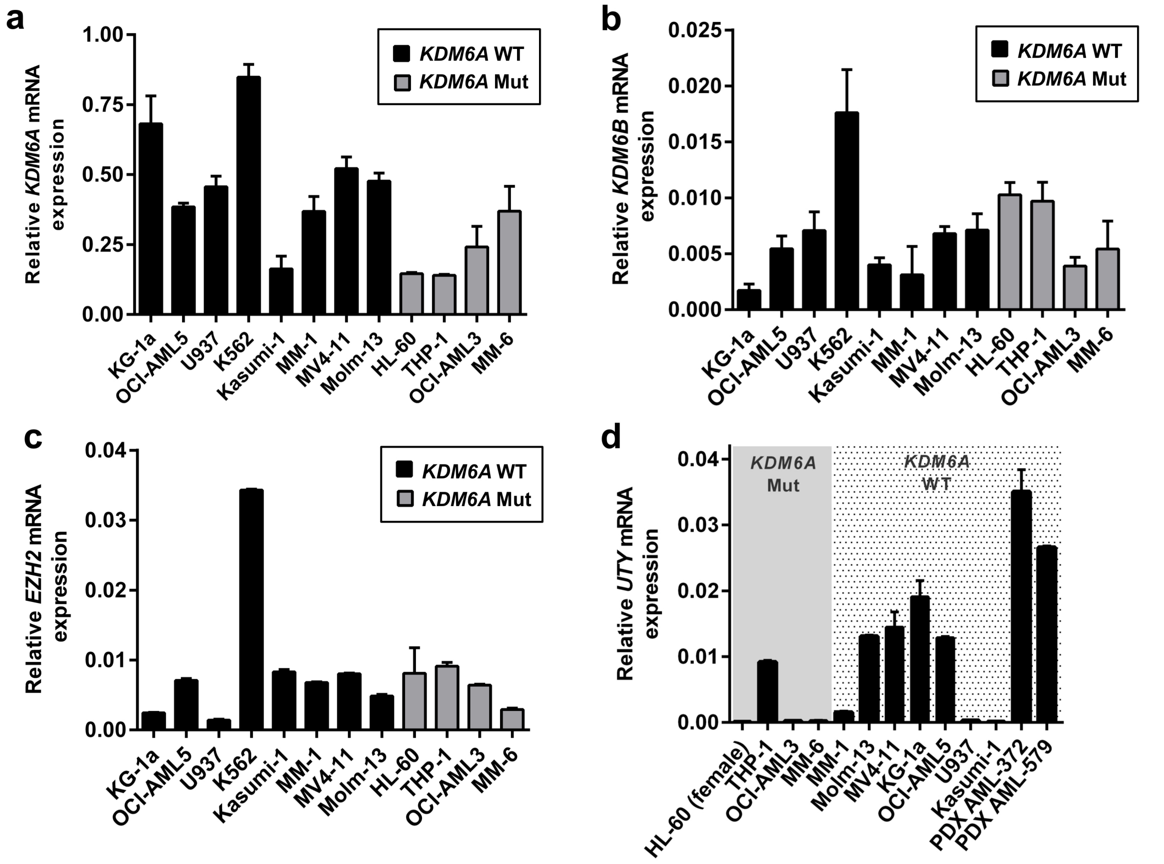

Supplementary Fig. 6, related to Fig. 3. No changes in *KDM6B* and *EZH2* mRNA expression, but loss of UTY in KDM6A mutant AML cells. a,b,c,d, qRT-PCR for *KDM6A* (**a**), *KDM6B* (**b**), *EZH2* (**c**), and *UTY* **(d)** in *KDM6A* WT and mutant AML cell lines. Two male PDX relapsed AML samples are additionally shown for *UTY* **(d)**. The mean ± s.d. relative to *GAPDH* for three experiments is shown.

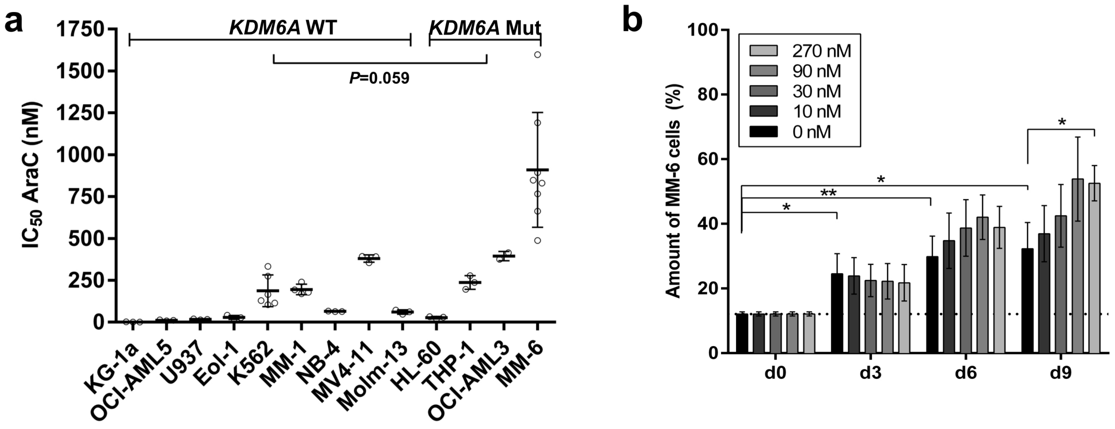

Supplementary Fig. 7, related to Fig 3. Competitive growth advantage of KDM6A mutant MM-6 cells under native conditions and AraC therapy. a, Comparison of AraC IC_{50} values between *KDM6A* WT and mutant AML cell lines. Mean ± s.d. from at least three independent experiments are shown. Unpaired, two-tailed Student's *t*-test; *P*=0.059. **b,** Changes in the amount of *KDM6A* mutant MM-6 cells relative to MM-1 cells for a time period of 9 days. MM-6 cells, labeled with Cell Violet, were mixed 1:9 with MM-1 (labeled with Cell Trace CFSE) cells on Day 0 and treated with increasing concentrations of AraC. Mean ± s.d. is given for three independent experiments. Unpaired, two-tailed Student's *t*-test; *$P<0.05$; **$P<0.01$.

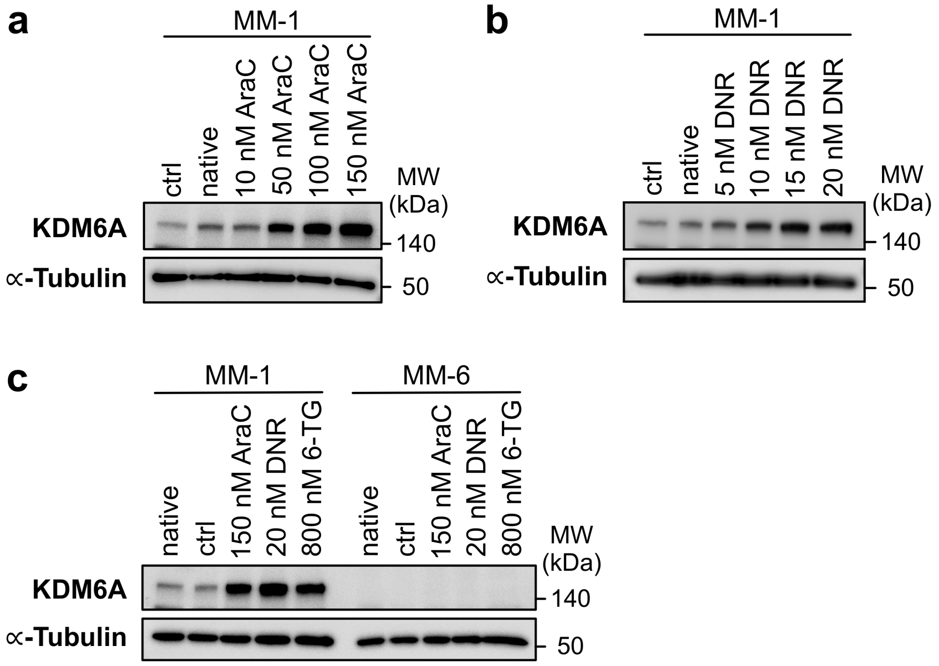

Supplementary Fig. 8. Upregulation of KDM6A protein expression after treatment with AraC, DNR and 6-TG. a, b, Immunoblot showing upregulation of KDM6A protein expression in MM-1 cells with increasing concentrations of AraC (**a**) and DNR (**b**) after 72h. **c,** Immunoblot showing upregulation of KDM6A protein expression in MM-1 cells after treatment with AraC, DNR or 6-TG for 72h. Applied concentrations were approximately the IC_{50} of each drug. *KDM6A* mutant MM-6 were used as a control. ctrl, DMSO control; MW, molecular weight; α-Tubulin, loading control.

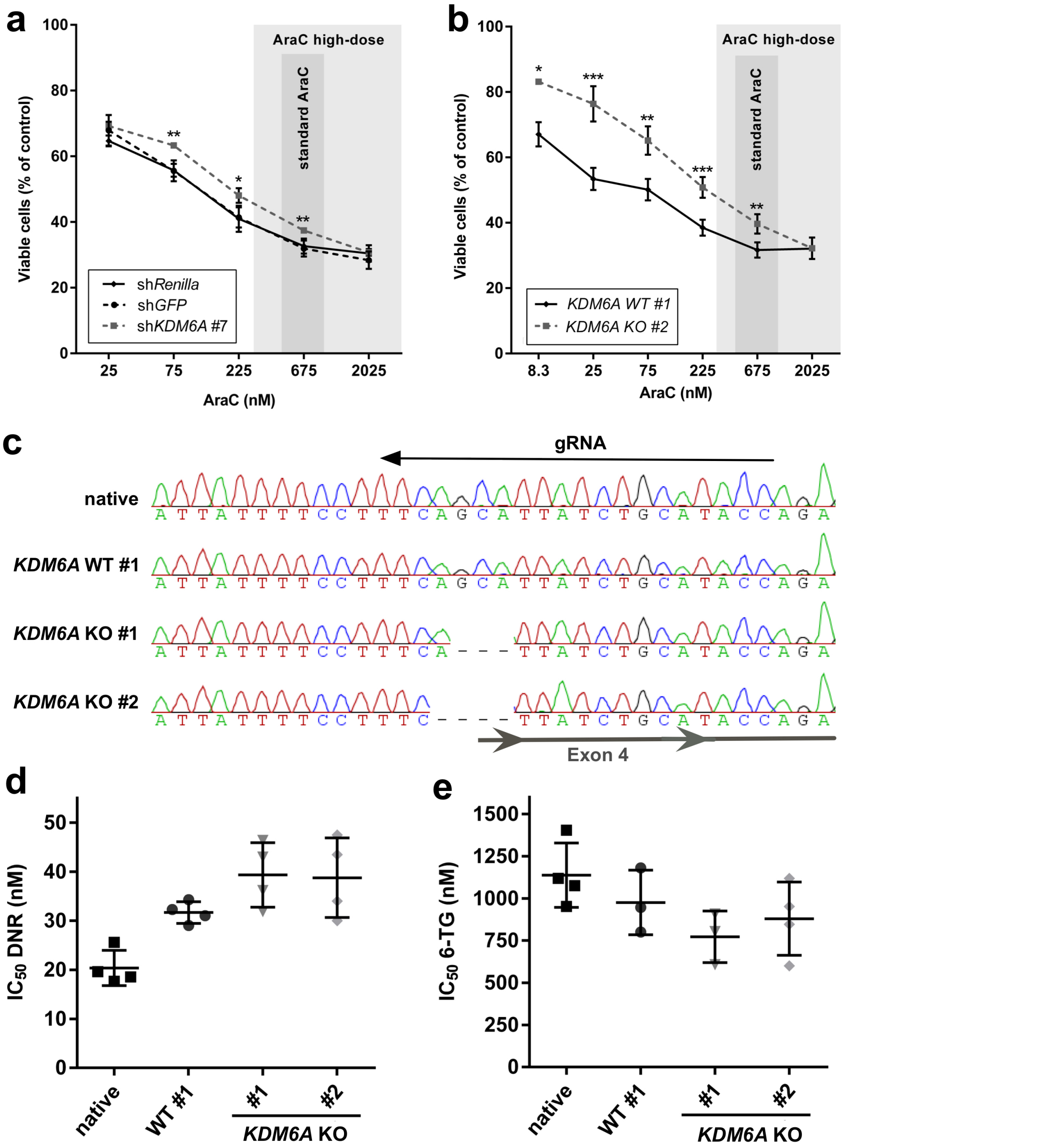

Supplementary Fig. 9, related to Fig. 4. Loss of *KDM6A* decreases sensitivity to AraC and DNR but not 6-TG. a,b, AraC dose-response analysis in K562 cells with modified KDM6A expression. Amount of viable cells after treatment with different AraC concentrations for 72h was compared between sh*Control* and sh*KDM6A* #7 cells (**a**) and between WT and *KDM6A* KO cells (**b**). The mean ± s.d. is given for technical duplicates of at least three independent experiments. The area shaded in dark grey (514 nM to 1028 nM) and light grey (226 nM-144 µM) indicates the range of steady-state plasma concentrations measured in patients during standard AraC (100-200mg/m^2) and after high-dose AraC (3000mg/m^2) treatment, respectively[4]. **c,** DNA sequencing chromatogram showing *KDM6A* frameshift mutations A112Vfs*12 in *KDM6A* KO K562 clone #1 and #2 compared with parental cells and a WT #1 clone. WT #1 clone was tested negative for *KDM6A* KO after CRISPR/Cas9 targeting. **d,e,** Comparison of IC_{50} values for DNR (**d**) and 6-TG (**e**) between K562 control cells (native and WT #1) and *KDM6A* KO clones #1 and #2 (72h treatment). Mean of IC_{50} values ± s.d. (*n*=3-4) are shown.

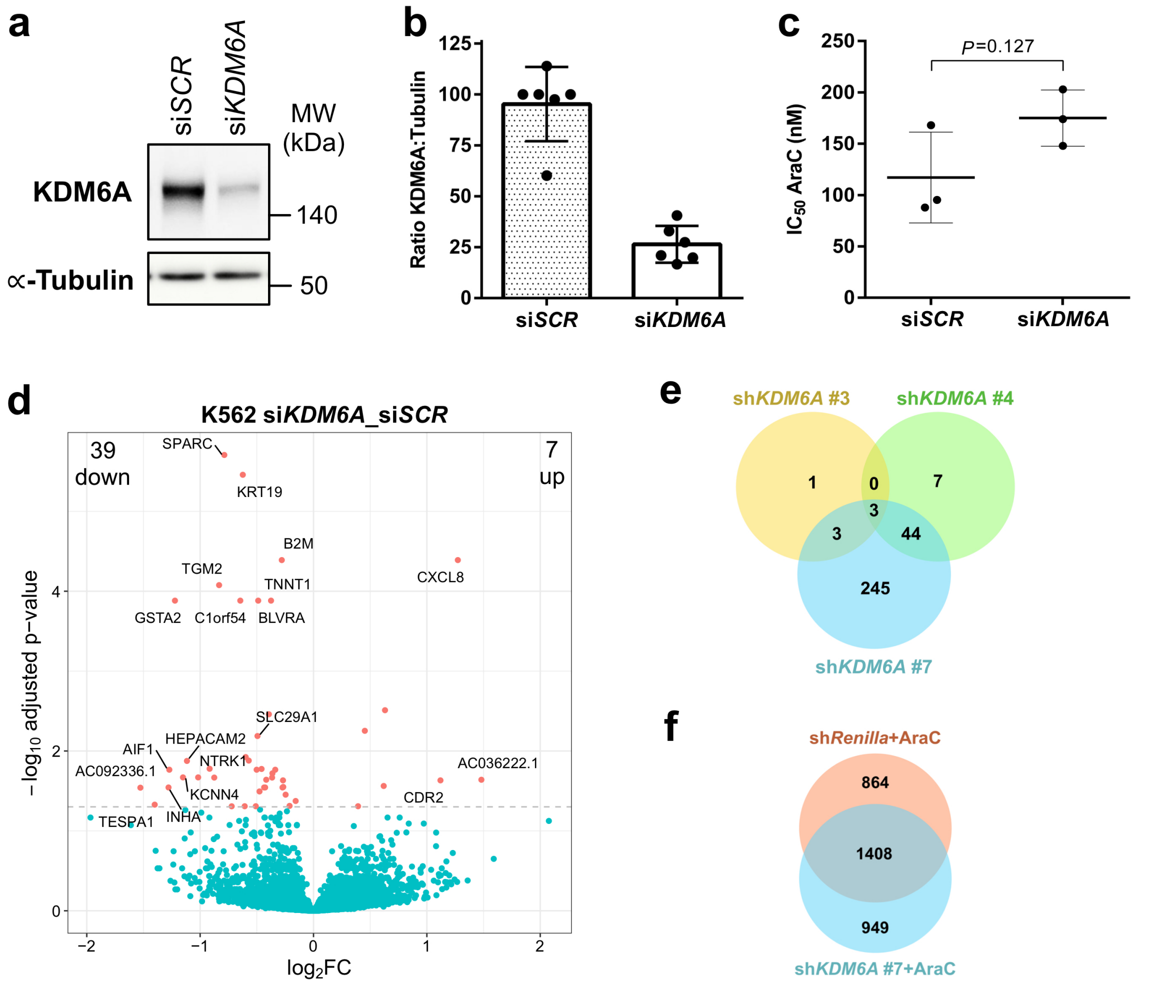

Supplementary Fig. 10, related to Fig. 7. Effect of *KDM6A* silencing regarding AraC resistance and target gene expression. a, Immunoblot showing KD of KDM6A expression in K562 cells after two rounds of transfection (2x72h) with siRNA against control (si*SCR*) and *KDM6A* (si*KDM6A*). MW, molecular weight; α-Tubulin, loading control. **b,** Knockdown efficiency of si*KDM6A* compared with control (si*SCR*) is shown for six independent experiments (mean ± s.d.). The ratio of KDM6A to α-Tubulin protein expression was calculated for each experiment and values were normalized to a control. **c,** Pro-proliferative effect of AraC treatment after *KDM6A* KD in K562 cells. 72h after second transfection, K562 cells (si*SCR* and si*KDM6A*) were treated with different concentrations of AraC for 72h. Mean of IC_{50} values from three independent experiments ± s.d. are shown. Unpaired, two-tailed Student's *t*-test; *P*=0.127. **d,** Volcano plot showing $\log_2$ fold change on the x-axis and adjusted *P* value on the y-axis for the differential gene expression between siRNA-mediated knockdown of *KDM6A* (si*KDM6A*) and control (si*SCR*) in K562 cells (*n*=6). Genes with adjusted *P* value <0.05 are highlighted in red and those with a $\log_2$ FC >1 or <-1 are labeled with the gene name. In addition, genes with adjusted *P* value < 0.01 and the gene *SLC29A1* are labeled. **e,** Overlap between differentially expressed genes (*P*<0.05) in three different sh*KDM6A* KD K562 cells. Differentially expressed genes of each sh*KDM6A* (#3, #4, and #7) compared to sh*GFP* control K562 cells are shown (*n*=6). **f,** AraC specific gene expression changes (*P*<0.05) in sh*KDM6A* #7 K562 cells compared with sh*Renilla* control K562 cells (*n*=6). Differentially

expressed genes in AraC (150 nM, 72h) treated samples were acquired by comparison with the respective untreated samples.

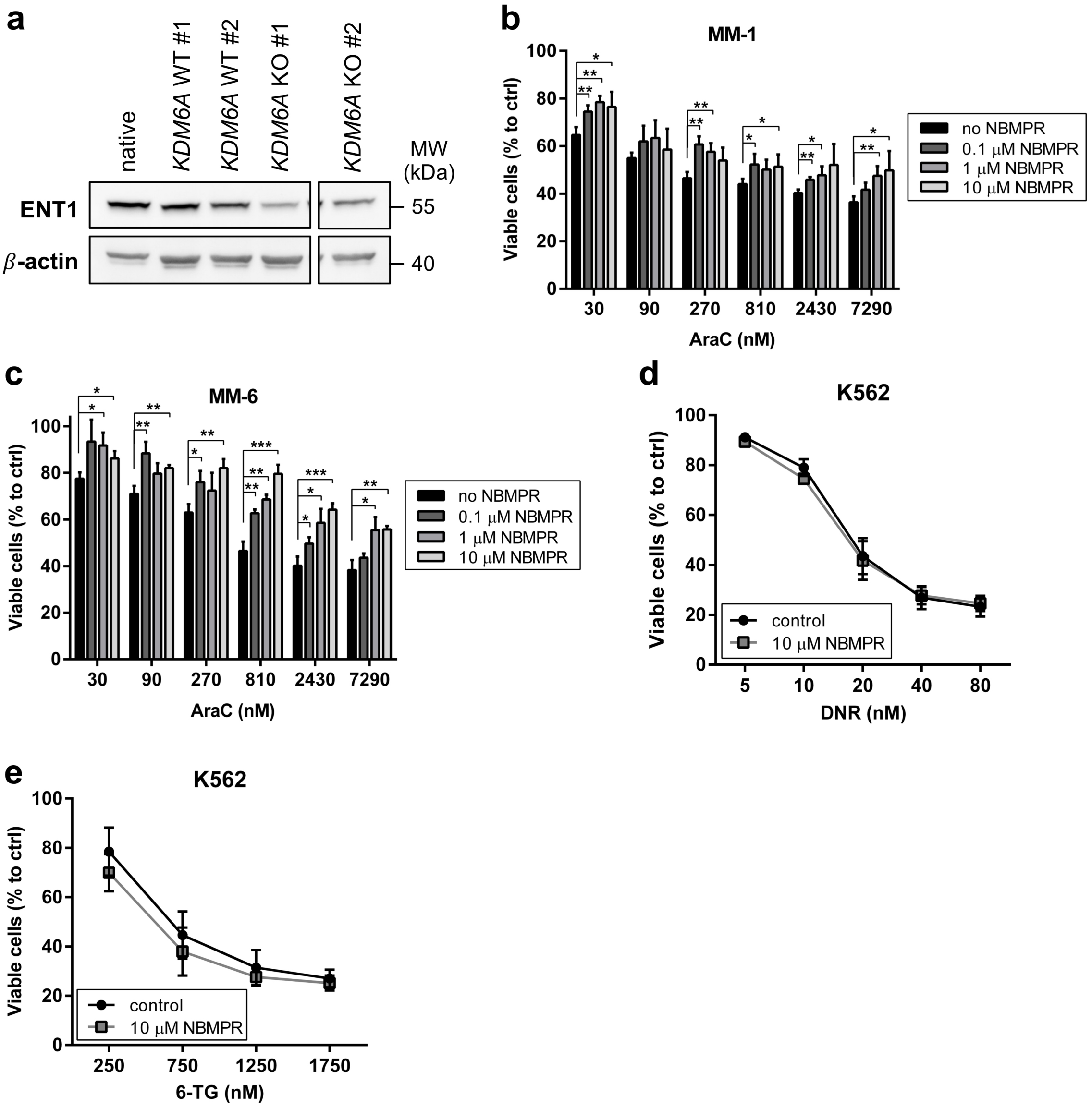

Supplementary Fig. 11, related to Fig. 7. Inhibition of ENT1 promotes resistance to AraC but not DNR and 6-TG. a, Immunoblot showing reduction of ENT1 expression in *KDM6A* KO K562 clones #1 and #2 compared with parental and WT clones. Both images, which have been separated, are from the same gel. MW, molecular weight; β-actin, loading control **b,c,** Inhibition of ENT1 by NBMPR increases the amount of viable cells during AraC treatment. MM-1 (**b**) and MM-6 (**c**) cells were treated with different AraC concentrations in combination with 0, 0.1, 1 and 10 μM of NBMPR for 72h and viable cells relative to untreated cells are shown. Mean ± s.d. are given for three independent experiments. Unpaired, two-tailed Student's *t*-test; *P<0.05; **P<0.01; ***P<0.001. **d,e,** Proliferative effect of ENT1 inhibition by NBMPR (10μM) compared with control (no inhibitor) in combination with different concentrations of DNR (**d**) and 6-TG (**e**) in K562 cells. The mean ± s.d. for three independent experiments is given.

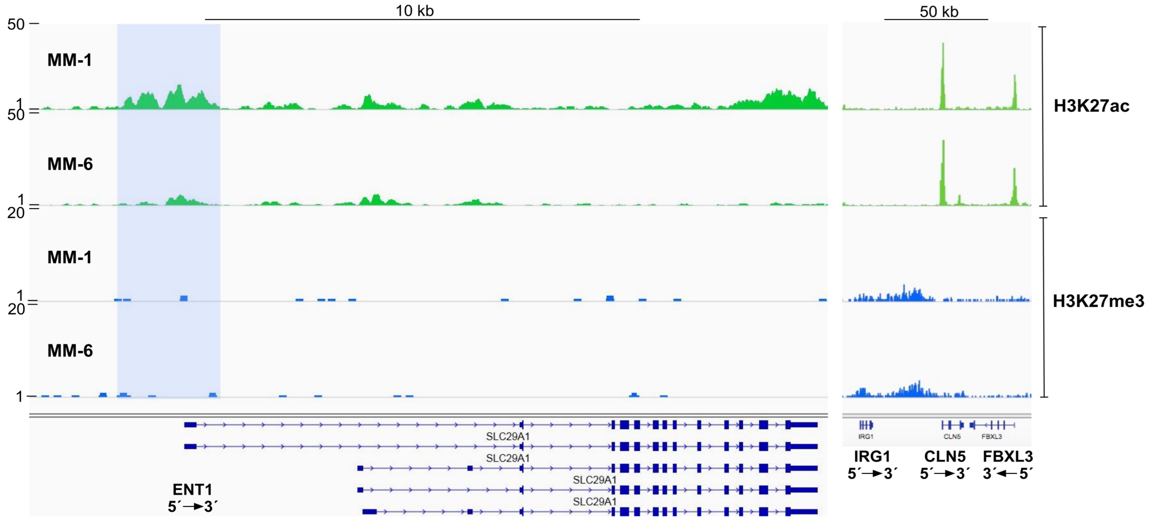

Supplementary Fig. 12, related to Fig. 7. *KDM6A* mutant MM-6 cells show decreased H3K27ac peaks at the *ENT1* locus. Genomic snapshot of H3K27ac and H3K27me3 ChIP-seq in *KDM6A* WT MM-1 and *KDM6A* mutant MM-6 cells at the *ENT1* locus. As control regions with no obvious changes, the *IRG1*, *CLN5*, and *FBXL3* loci are shown. The light blue tinted box highlights the differential peaks of interest.

Supplementary Table 1. Summary of analyzed cell lines. *KDM6A* exon deletions, detected by quantitative MLPA and CytoScan HD Array hybridization analysis, are listed together with name, cell type, and gender.

Name	Cell Type	Gender	*KDM6A* exon deletion
AP-1060	AML	male	-
Eol-1	AML	male	-
F-36P	AML	male	-
FKH-1	AML	male	-
GF-D8	AML	male	-
HEK293T	embryonic kidney	female	n.a.
HL-60	AML	female	del exon 5-6
HNT-34	AML	female	-
HT-93	AML	male	-
K562	CML in blast crisis	female	-
Kasumi-1	AML	male	-
Kasumi-3	AML	male	-
KG-1a	AML	male	-
M-07e	acute megakaryoblastic leukemia	female	-
ME-1	AML	male	-
MEGAL	acute megakaryoblastic leukemia	n.s.	-
MKPL-1	acute megakaryoblastic leukemia	male	-
MONO-MAC-1 (MM-1)	acute monocytic leukemia	male	-
MONO-MAC-6 (MM-6)	acute monocytic leukemia	male	del exon 3-10
Molm-13	AML	male	-
Molm14 (sister Molm-13)	AML	male	-
Molm16	AML	female	-
MUTZ-2	AML	male	-
MUTZ-3	acute myelomonocytic leukemia	male	-
MV4-11	acute monocytic leukemia	male	-
NB-4	acute promyelocytic leukemia	female	n.a.
OCI-AML1	AML	female	-
OCI-AML3	AML	male	del exon 3-4
OCI-AML4	AML	female	-
OCI-AML5	AML	male	-
OCI-AML6	sAML	female	-
OCI-M1	AML	n.s.	-
PLB-985 (sister HL-60)	AML	female	-
SKM-1	AML	male	-
SKNO-1	AML	male	-
TF-1	erythroleukemia	male	-
THP-1	acute monocytic leukemia	male	del exon 1-16
U-937	histiocytic lymphoma	male	-
UCSD-AML1	AML	female	-
UOC-M1	AML	male	-
UT-7	AML	male	-
YNH-1	AML	male	-

n.a., not analyzed; n.s., not specified.

Supplementary methods

CFSE staining

MM-1 and MM-6 cells were labeled with the respective Cell Trace CFSE and Violet Cell Proliferation Kit (Thermo Fisher Scientific). Cell staining was performed as previously described[5]. Cells were then mixed in a 9:1 ratio and incubated with different AraC concentrations. Every 72h, a small portion was analyzed by flow cytometry (FACSCanto II, BD Bioscience, Franklin Lakes, NJ, USA) using 1 µg/mL propidium iodide (Sigma Aldrich, St. Louis, MO, USA) to exclude dead cells.

DNA constructs

Human KDM6A with N-terminal HA was amplified from pCMV-HA-UTX (Addgene #24168), and cloned into the pcDNA6/V5-His A vector (Thermo Fisher Scientific) using the In-Fusion HD Cloning Plus Kit (Takara Bio, Saint-Germain-en-Laye, France). KDM6A mutants were generated using the QuikChange II XL Site-Directed Mutagenesis Kit (Agilent Technologies, Santa Clara, CA, USA) and correct sequence was confirmed by Sanger sequencing.

Immunoblotting

Immunoblotting was performed as previously described[1]. Following antibodies were used: anti-KDM6A (#33510, Cell Signaling Technology, Danvers, MA, USA), anti-HA-Tag (#2367**,** Cell Signaling), anti-ENT1 (STJ96396, St John's Laboratory, London, UK), anti-β-actin (A5441, Sigma Aldrich), anti-α-Tubulin (T6199, Sigma Aldrich), anti-H3 (ab1791, Abcam, Cambridge, UK), anti-H3K27me1 (07-448, Merck Millipore, Billerica, MA, USA), anti-H3K27me2 (07-452, Merck Millipore), anti-H3K27me3 (07-449, Merck Millipore)**.** Western blot signals were quantified using ImageJ version 1.50d and relative levels were normalized to loading control.

qRT-PCR analysis

Total RNA was isolated using the RNeasy Mini and RNase-Free DNase Kit (Qiagen). cDNA was synthesized by reverse transcription with the ThermoScript RT-PCR System (Thermo Fisher Scientific, Waltham, MA, USA) using 1 µg of total RNA as input. qRT-PCR assays were performed with QuantiTect SYBR Green PCR Kit (Qiagen) using 500 ng of cDNA and the following primers: *KDM6A*[6]; *KDM6B*[6]; *GAPDH*[6]; *EZH2*: fwd 5′-CCCTGACCTCTGTCTTACTTGTGGA-3′, rev 5′-ACGTCAGATGGTGCCAGCAATA-3′; *ENT1*[7], and *UTY*: fwd 5′-TTAGCCTGACAGTCGAGGAAA-3′, rev 5′-GTAGGGTCTTCGTTCTGGCG-3′. Reactions were run on a LightCycler480 II (Roche, Basel, CH). Fold changes were calculated using the $\Delta\Delta C_t$ method and normalized against *GAPDH* expression.

CRISPR/Cas9 gene editing

KDM6A specific gRNA (5′-GGTATGCAGATAATGCTGAA-3\`) was cloned into pSpCas9(BB)-2A-GFP (PX458), a gift from Feng Zhang (Addgene #48138). The gRNA was designed using Benchling (Biology Software, 2018). 48h after nucleofection with 1-2 µg plasmid, GFP positive cells were enriched by single-cell sorting into 96-well V-bottom

plates with FACSVantage SE (BD Bioscience). Cells were cultured until colonies were readily visible. Cell lysis, PCR on lysates and restriction digest were performed as previously described with minor modifications[8]. Briefly, for gDNA isolation cells in 96-well plate were washed with PBS (2x), resuspended in 50 µL/well lysis buffer (50 mM TRIS/HCl pH 7.5, 10 mM CaCl2, 1.7 µM SDS, 50 µg/ml Proteinase K), frozen at -80°C for 30 min, incubated at 56°C for 3 h, and 85°C for 30 min. 2.5 µL/well of the resulting lysate were subjected to PCR using forward 5´-GGGGTTAGCCTAGATGCTGTTC-3`, and reverse primer 5`-ATTGGCAATAATCTGCCCAAAACA-3´. KO clones were identified by restriction-fragment length polymorphism (RFLP) analysis of PCR products using HpyF10VI (Thermo Fisher Scientific). Sanger sequences were analyzed and aligned with Geneious 8.1.7 (Biomatters Ltd, Auckland, New Zealand) software and Benchling.

DNA sequencing

gDNA was isolated with the QIAamp DNA Blood Mini Kit (Qiagen) manually or using a Qiacube instrument (Qiagen). Mutation E1325X was verified by Sanger sequencing both DNA strands of PCR-amplified gDNA using 3500/3500xL Genetic Analyzer (Applied Biosystems). First, PCR was performed with forward primer 5`-CACGGATGAGGAAATTGACTCC-3` and reverse primer 5`-GGCATCTGTGTACATCTAGATTGTTCTTAG-3` followed by a second PCR and sequence analysis with forward primer 5`-CAGGCCTGCTGAGCATTG-3`and reverse primer 5`-GAAACCAACAGTGGAGAGGG-3. Targeted, multiplexed amplicon resequencing covering the entire open-reading frame of *KDM6A* and mutational hotspots/entire open-reading frame of genes known to be recurrently mutated in myeloid malignancies was performed as described previously[9].

Transfection

HEK293T cells were transiently transfected using the calcium-phosphate precipitation method. Briefly, 13µg plasmid DNA in 450µL H_2O was mixed with 50µL 2M $CaCl_2$. The resulting mixture was slowly added to 500µL 2x HBS buffer (Sigma Aldrich), incubated for 4 min and added drop wise to a 10cm dish containing 90% confluent cells. For MG-132 treatment, 42h after transfection, cells were incubated with fresh media containing 50 µM MG-132 (Sigma Aldrich) for 6h. Suspension cells were transfected using the Amaxa nucleofection system with Nucleofector Kit V and preprogrammed settings (K562: T-016; MM-1: T-030/T-036; THP:1 U-001) on the Amaxa Nucleofector II device (Lonza Group AG, Basel, CH).

***In* vivo therapy trial**

Patient-derived xenograft (PDX) cells expressing firefly luciferase were established as previously described[9]. $5*10^5$ AML 491 or AML 393 cells were injected i.v. into groups (n=4; n=3 for AML 491 treated with AraC) of NSG mice (NOD scid gamma, The Jackson Laboratory, Bar Harbour, ME, USA), and tumor growth was regularly monitored by bioluminescence imaging (BLI) as previously described[9]. At defined imaging signals, mice were treated with a combination of cytarabine (100 mg/kg, i.p., days 1-4 and 14-17) and DaunoXome (1 mg/kg, i.v., days 1, 4, 14, 17). 28 days after start of therapy, BLI was performed and increase in BLI signals relative to day 0 were calculated. No randomization was used. All animal trials were performed in accordance with the current ethical standards

of the official committee on animal experimentation (Regierung von Oberbayern, number 55.2-1-54-2531-95-2010).

siRNA

K562 cells were nucleofected with Stealth siRNA (Thermo Fisher Scientific) against *KDM6A* (HS111232; 5'-GCAAAUGUUCCAGUGUAUAGGUUUA-3`) or negative control siRNA (si*SCR*; low GC; 12935200). After 72h, cells were nucleofected for a second time and incubated for 72h, after which cells were used for subsequent analysis.

Lentiviral transduction

Lentiviruses expressing *KDM6A*-targeting shRNA #3 (5′-TACTTGAATAGCACCTTCCGA-3′), #4 (5′-TTTAATGGCATCCTGAGGCTG-3′), #7 (5′-TTTATCAATAGACTGCCTGTA-3′) or control shRNA targeting Renilla (5′-TAGATAAGCATTATAATTCCT-3′) or eGFP (5′-CAGCCACAACGTCTATATCAT-3′) were generated by cloning into the pCDH-EF1α-MCS-T2A-copGFP vector (SBI, CD521A-1). dsRED-miR30 fragment (TRMPVIR vector; Addgene #27994) was amplified using primers carrying MfeI and SalI as 5' and 3' restriction sites. The 22mer shRNA target sequences have been synthetized as part of 110bps ssDNA oligos (Eurofins Scientific, Luxembourg), annealed and cloned into the vector. To enhance the shRNA expression, the EF1α promoter was replaced by the viral promoter SFFV. Production of lentiviral particles and transduction was performed as previously described[10,11]. After a few days, transgene positive cells were enriched in two consecutive rounds by flow cytometry using FACSVantage SE (BD Bioscience).

MLPA and numerical aberrations

MLPA analysis for *KDM6A* exon deletions was carried out as previously described[1]. To identify numerical *KDM6A* aberrations in AML cell lines, CytoScan HD Array (Affymetrix, Santa Clara, CA, USA) hybridization analysis were performed. The DNA was prepared using the Qiagen Gentra Puregene Kit (Qiagen). Labeling, hybridization and scanning were performed according to the manufacturer´s protocol. Data were analyzed using the Chromosome Analysis Suite software version 2.0.1.2 (Affymetrix).

PiggyBac Construct

To generate the PiggyBac (PB) donor vector harboring the doxycycline inducible expression cassette, the tet-3xFLAG-AsiSI-NotI-IRES-DsRed-Express-M2rtTA-P2A-PuroR cassette was amplified from pSBtet-3xFLAG-IRES-DsRed-Express-PuroR[12] using primers PB.fwd (5`-acggggaaaaggcctccaGGTCCGCTATCTAGACGA-3) and PB.rev (5`-aagccataccaatgggccGCTAGTAGGCCCAGCT-3) with overhangs for subsequent DNA assembly. The insert Cuo-MCS-IRES-GFP-EF1α-CymR-PuroR was excised from the PiggyBac expression vector PB-Cuo-MCS-IRES-GFP-EF1α-CymR-Puro (System Biosciences, #PBQM812A-1) using ApaI and SfiI (Thermo Fisher Scientific). The 3xFLAG-AsiSI-NotI-IRES-DsRed-Express-M2rtTA-P2A-PuroR fragment was then cloned into the ApaI and SfiI linearized PiggyBac backbone using the NEBuilder HiFi DNA Assembly Master Mix (New England BioLabs) according to the manufacturer's

instructions generating the vector pPBtet-3xFLAG-IRES-DsRed-Express-PuroR. Human full length KDM6A without the N-terminal HA Tag was amplified from pcDNA6 HA KDM6A with overhangs for subsequent DNA assembly. The KDM6A fragment was cloned into the BmtI and NotI linearized pPBtet-3xFLAG-IRES-DsRed-Express-PuroR vector (3xFLAG was removed by linearization) using the In-Fusion HD Cloning Plus Kit (Takara Bio, Saint-Germain-en-Laye, France).

Generation of inducible PiggyBac KDM6A cell lines

To generate stable cell lines carrying doxycycline inducible KDM6A, cells were first nucleofected with equimolar amounts of pPBtet-KDM6A-IRES-DsRed-Express-PuroR and PiggyBac transposase (Biocat, PB200PA-1). Two days after transfection, cells were subjected to puromycin selection (2 µg/mL) for 3 days. Viable cells were enriched by single-cell sorting into 96-well V-bottom plates with FACSVantage SE (BD Bioscience). Cells were cultured with puromycin (2 µg/mL) until colonies were readily visible. To screen for successful KDM6A re-expression, clones were treated with doxycycline (0.5 µg/mL) for 48h and inducible KDM6A expression was analyzed by Western Blot.

Library Preparation and Sequencing of Cell Lines

To construct bulk libraries from mRNA, a protocol adapted from the SCRB-seq method was used[13]. Briefly, 50 ng mRNA was reverse transcribed using Maxima H Minus Reverse Transcriptase and tagged with sample-specific barcodes and unique molecular identifiers (UMIs). Only 2 µM of the E3V6NEXT was used. Samples were pooled and purified by SPRI beads, followed by an Exonuclease I treatment. Full-length cDNA was pre-amplified by single primer PCR for 10 cycles with the modification of using KAPA Hifi 2x ready mix (KAPA Biosystems). For the Nextera XT kit, 4 ng of cDNA was used as input and library preparation performed according to the manufacturer's protocol, with the exception of using a custom i5 primer. Sequencing was performed on an Illumina HiSeq 1500, flow cell with single end layout utilizing the standard Illumina sequencing and index primers. Sample reads were sequenced using 50 cycles and the UMI sequence using 16 cycles. To obtain expression data the raw fastq files were processed by the zUMIs pipeline using default parameters[14]. Mapping to the human reference genome hg38 was performed by STAR[15] (version 2.5.2b) and the gene annotation GRCh38.84 was taken from Ensembl. Differential expression analysis was performed using limma[16]. Genes with a read count below 10 in all samples were filtered out and library sizes scaled using the package edgeR[17]. Count data was transformed to log2-counts per million and the mean variance calculated to compute the precision weights. In order to increase statistical power empirical Bayes moderation was applied and false discovery rate (FDR) calculated by the Benjamini-Hochberg procedure.

Sequencing of Primary Patient Data Analysis

Sequencing of mRNA was performed as previously described[18] using the TruSeq RNA Sample Preparation protocol, followed by sequencing on a HiSeq 2000 Instrument (Illumina). Primary patient data was mapped to the human reference genome hg19 using 2-pass mapping with the RNA-Seq aligner STAR (version 2.4.0.1)[15], allowing only one alignment per read. Otherwise default parameters were used and the gene annotation GRCh37.75 taken from Ensembl. Mapped reads were then assigned to genes (Ensembl annotation version GRCh37.75) using the function featureCounts from the R package

Rsubread[19]. Subsequently, count tables for reads per genes were generated and normalized using the functions estimateSizeFactors and estimateDispersions of the DESeq2 package[20]. The three groups, *KDM6A*-up, *KDM6A*-down and *KDM6A*-same were defined as a change in expression between Diagnosis and Relapse of above or below 20% respectively.

DNA Methylation Analysis

KDM6A methylation status was determined based on 344 previously described AML patients[2]. This data is accessible via the Gene Expression Omnibus database repository (GSE18700). Clinical correlation of KDM6A methylation status and overall survival was determined using the Leukemia Gene Atlas version 2.1.0[21].

ChIP-seq of Histone Modifications

ChIP-seq reads were aligned to the human reference genome (hg38) using bowtie[22] with "-q-n2--best --chunkmbs2000- p 32 -m1" options. bigWig files were generated from homer tag directories using makeBigWig.pl. 1-2 million cross-linked cells (1% formaldehyde, 10min at RT) were lysed in 100 µL Buffer-B (50 mM Tris-HCl, pH 8.0, 10 mM EDTA, 1%SDS, 1x protease inhibitors) and sonicated until most of the DNA fragments were 200-500 base pairs long (Covaris S220; 4°C, 30min, duty cycle 2%, 105 Watts). Lysates were then centrifuged (10min, 4°C, 12000g) and supernatant was diluted with 900 µL Buffer-A (10 mM Tris-HCl, pH 7.5, 1 mM EDTA, 0.5 mM EGTA,1% Triton X-100, 0.1% SDS, 0.1% Sodiumdeoxycholate, 140 mM NaCl, 1x protease inhibitors). 150 µL of sonicated chromatin was then incubated on a rotating wheel (3h, 4°C) with 2 µg of antibody conjugated to 10 µL Dynabeads. Antibodies used: H3K27ac (Diagenode; Pab-174-050), H3K27me3 (Pab-069-050). Beads were washed 4x with Buffer-A and once with Buffer-C (10 mM Tris-HCl, pH 8.0, 10 mM EDTA). Beads were incubated with 70 µL elution buffer (0.5% SDS, 300 mM NaCl, 5 mM EDTA, 10 mM Tris HCl pH 8.0) containing 2 µL of RNase (10mg/ml) for 30 min (37°C, 900rpm) and then with 2 µL of Proteinase K (20mg/ml) for 1h (55°C) and 8h (65°C) and supernatant was transferred to a new tube. Another 30 µL of elution buffer was added to the beads for 1 minute and eluates were combined and incubated with another 1 µL of Proteinase K for 1h at 55°C. Finally, DNA was purified with SPRI AMPure XP beads (Beckman Coulter), sample-to-beads ratio 1:2. Purified DNA was used as input for library preparation with NEBNext Ultra II DNA Library Prep Kit for Illumina (Biolabs, E7645S) and processed according to the manufacturer´s instructions. Libraries were quality controlled by Qubit and Agilent DNA Bioanalyzer analysis. Deep sequencing was performed on HiSeq1500/2500 according to the standard Illumina protocol for 50bp single-end reads. The dataset is publicly available under GSE128262.

[1] P.A. Greif, L. Hartmann, S. Vosberg, S.M. Stief, R. Mattes, I. Hellmann, et al., Evolution of Cytogenetically Normal Acute Myeloid Leukemia During Therapy and Relapse: An Exome Sequencing Study of 50 Patients., Clin. Cancer Res. 24 (2018) 1716–1726. doi:10.1158/1078-0432.CCR-17-2344.

[2] M.E. Figueroa, S. Lugthart, Y. Li, C. Erpelinck-Verschueren, X. Deng, P.J. Christos, et al., DNA methylation signatures identify biologically distinct subtypes in acute myeloid leukemia, Cancer Cell. 17 (2010) 13–27. doi:10.1016/j.ccr.2009.11.020.

[3] W. Liu, Y. Xie, J. Ma, X. Luo, P. Nie, Z. Zuo, et al., IBS: an illustrator for the presentation and visualization of biological sequences., Bioinformatics. 31 (2015) 3359–3361. doi:10.1093/bioinformatics/btv362.

[4] A.P. Early, H.D. Preisler, H. Slocum, Y.M. Rustum, A pilot study of high-dose 1-beta-D-arabinofuranosylcytosine for acute leukemia and refractory lymphoma: clinical response and pharmacology., Cancer Res. 42 (1982) 1587–94. http://www.ncbi.nlm.nih.gov/pubmed/6949642 (accessed June 6, 2018).

[5] A.B. Lyons, K. V. Doherty, Flow Cytometric Analysis of Cell Division by Dye Dilution, Curr. Protoc. Cytom. (2004) 9.11.1-9.11.10.

[6] W. Jiang, D. Zhang, N. Bursac, Y. Zhang, WNT3 is a biomarker capable of predicting the definitive endoderm differentiation potential of hESCs., Stem Cell Reports. 1 (2013) 46–52. doi:10.1016/j.stemcr.2013.03.003.

[7] H. Kitao, Y. Morodomi, S. Niimi, M. Kiniwa, K. Shigeno, K. Matsuoka, et al., The antibodies against 5-bromo-2'-deoxyuridine specifically recognize trifluridine incorporated into DNA., Sci. Rep. 6 (2016) 25286. doi:10.1038/srep25286.

[8] C.B. Mulholland, M. Smets, E. Schmidtmann, S. Leidescher, Y. Markaki, M. Hofweber, et al., A modular open platform for systematic functional studies under physiological conditions, Nucleic Acids Res. 43 (2015). doi:10.1093/nar/gkv550.

[9] B. Vick, M. Rothenberg, N. Sandhöfer, M. Carlet, C. Finkenzeller, C. Krupka, et al., An Advanced Preclinical Mouse Model for Acute Myeloid Leukemia Using Patients' Cells of Various Genetic Subgroups and In Vivo Bioluminescence Imaging, PLoS One. 10 (2015) e0120925. doi:10.1371/journal.pone.0120925.

[10] N. Terziyska, C. Castro Alves, V. Groiss, K. Schneider, K. Farkasova, M. Ogris, et al., In vivo imaging enables high resolution preclinical trials on patients' leukemia cells growing in mice., PLoS One. 7 (2012) e52798. doi:10.1371/journal.pone.0052798.

[11] S. Ebinger, E.Z. Özdemir, C. Ziegenhain, S. Tiedt, C. Castro Alves, M. Grunert, et al., Characterization of Rare, Dormant, and Therapy-Resistant Cells in Acute Lymphoblastic Leukemia., Cancer Cell. 30 (2016) 849–862. doi:10.1016/j.ccell.2016.11.002.

[12] C.B. Mulholland, J. Ryan, W. Qin, M.D. Bartoschek, F.R. Traube, S. Bultmann, et al., TET1 drives global DNA demethylation via DPPA3-mediated inhibition of maintenance methylation, BioRxiv. 321604 (2018). doi:10.1101/321604.

[13] T. Soumillon, M. Cacchiarelli, D. Semrau, S. van Oudenaarden, A. Mikkelsen, Characterization of directed differentiation by highthroughput single-cell RNA-seq., BioRxiv. (2014).

[14] S. Parekh, C. Ziegenhain, B. Vieth, W. Enard, I. Hellmann, zUMIs - A fast and flexible pipeline to process RNA sequencing data with UMIs, Gigascience. 7 (2018). doi:https://doi.org/10.1093/gigascience/giy059.

[15] A. Dobin, C.A. Davis, F. Schlesinger, J. Drenkow, C. Zaleski, S. Jha, et al., STAR: ultrafast universal RNA-seq aligner, Bioinformatics. 29 (2013) 15–21. doi:10.1093/bioinformatics/bts635.

[16] M.E. Ritchie, B. Phipson, D. Wu, Y. Hu, C.W. Law, W. Shi, et al., limma powers

differential expression analyses for RNA-sequencing and microarray studies., Nucleic Acids Res. 43 (2015) e47. doi:10.1093/nar/gkv007.
[17] M.D. Robinson, D.J. McCarthy, G.K. Smyth, edgeR: a Bioconductor package for differential expression analysis of digital gene expression data., Bioinformatics. 26 (2010) 139–40. doi:10.1093/bioinformatics/btp616.
[18] L. Hartmann, S. Dutta, S. Opatz, S. Vosberg, K. Reiter, G. Leubolt, et al., ZBTB7A mutations in acute myeloid leukaemia with t(8;21) translocation., Nat. Commun. 7 (2016) 11733. doi:10.1038/ncomms11733.
[19] Y. Liao, G.K. Smyth, W. Shi, featureCounts: an efficient general purpose program for assigning sequence reads to genomic features., Bioinformatics. 30 (2014) 923–30. doi:10.1093/bioinformatics/btt656.
[20] M.I. Love, W. Huber, S. Anders, Moderated estimation of fold change and dispersion for RNA-seq data with DESeq2., Genome Biol. 15 (2014) 550. doi:10.1186/s13059-014-0550-8.
[21] K. Hebestreit, S. Gröttrup, D. Emden, J. Veerkamp, C. Ruckert, H.-U. Klein, et al., Leukemia gene atlas--a public platform for integrative exploration of genome-wide molecular data, PLoS One. 7 (2012) e39148. doi:10.1371/journal.pone.0039148.
[22] B. Langmead, C. Trapnell, M. Pop, S.L. Salzberg, Ultrafast and memory-efficient alignment of short DNA sequences to the human genome, Genome Biol. 10 (2009) R25. doi:10.1186/gb-2009-10-3-r25.
